# Elucidation of the Verrucofortine Biosynthetic Pathway Enables Identification of a Cyclodipeptide Prenyltransferase with High Catalytic Efficiency

**DOI:** 10.1021/acs.jafc.6c01966

**Published:** 2026-03-03

**Authors:** Hui-Ling Wei, Li-Dan Pang, Xiao-Ling Chen, Shu-Ming Li

**Affiliations:** 9377 Philipps-Universität Marburg, Fachbereich Pharmazie, Institut für Pharmazeutische Biologie und Biotechnologie, Robert-Koch-Straße 4, 35037 Marburg, Germany

**Keywords:** cyclodipeptide, Penicillium polonicum, prenyltransferase, verrucofortine biosynthesis

## Abstract

*Penicillium polonicum*, a common spoilage fungus affecting stored grains, nuts, and dried fruits, produces diverse secondary metabolites, including the indole-diketopiperazine derivative verrucofortine. Here, we elucidate its biosynthetic pathway through targeted gene deletion in a constructed low-metabolite-background strain and biochemical characterization with recombinant proteins. A nonribosomal peptide synthetase (VftPS) is responsible for assembling the cyclodipeptide scaffold, which is then modified by the prenyltransferase VftPT and the acetyltransferase VftAT. Notably, VftPT shows higher catalytic efficiency toward all the tested tryptophan-containing cyclodipeptides for *C3ß*-prenylation at the indole ring than its known homologues, highlighting its potential for biocatalysis and small molecule modification.

## Graphical abstract

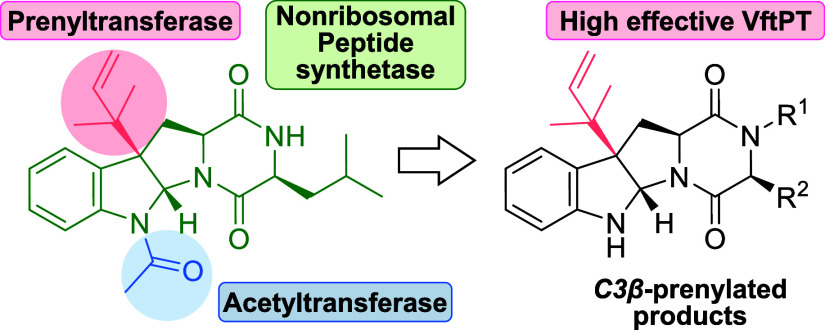

## 1 Introduction

*Penicillium polonicum* is a widespread fungus and commonly found in soil, decaying vegetation, and stored agricultural products, including grains, dried fruits, nuts, and animal feed.^1–4^ Contamination with this fugus under suboptimal postharvest conditions, especially with high humidity and moderate temperatures, can lead to substantial quality deterioration, discoloration, and nutrient loss.^5^ Like most fungi, *P. polonicum* also produce a variety of secondary metabolites including mycotoxins harmful for animals and human being.^6^ On the other hand, mycotoxins can serve as potential biologically and pharmacologically interesting molecules and the enzymes involved in their biosynthesis can be used as useful biocatalysts.^7^

Among the metabolites produced by *P. polonicum*, verrucofortine (**1**), also known as fructigenine B in *Penicillium fructigenum*, represents a typical indole alkaloid and features a Trp-Leu-derived 2,5-diketopiperazine scaffold.^8,9^ This compound is a member of pyrrolo-[2,3-*b*]-indole type metabolites, a distinctive class of fungal indole alkaloids characterized by a 2,5-diketopiperazine core fused with an indole-derived pyrrolo framework. Their biosynthesis generally begins with the formation of a cyclodipeptide precursor via a nonribosomal peptide synthetase (NRPS) or cyclodipeptide synthase (CDPS) pathway, which condense two free or tRNA-activated amino acids—often tryptophan and another residue—into the 2,5-diketopiperazine scaffold.^10^ This precursor subsequently undergoes extensive postassembly modifications, such as prenylation,^11,12^ methylation,^11^ nucleobase transfer,^13^ dimerization,^14^ and hydroxylation,^15^ for construction of the characteristic pyrrolo-[2,3-*b*]-indole scaffold with high diversity and stereochemical complexity (Figure 1).^16^ Prenylation, e.g. on indole of tryptophan-containing cyclodipeptide backbones, is a crucial tailoring reaction for structure diversity and biological activity.^17^ To continue our studies on the biosynthesis of prenylated cyclodipeptide derivatives, we elucidated the biosynthetic pathway of verrucofortine in *P. polonicum* through a combination of genome mining, targeted gene disruption, and biochemical characterization. Kinetic analysis further revealed that VftPT is a highly efficient enzyme compared to most of the previously reported homologues, highlighting its potential utility in biocatalysis and synthetic biology.

**Figure 1:**
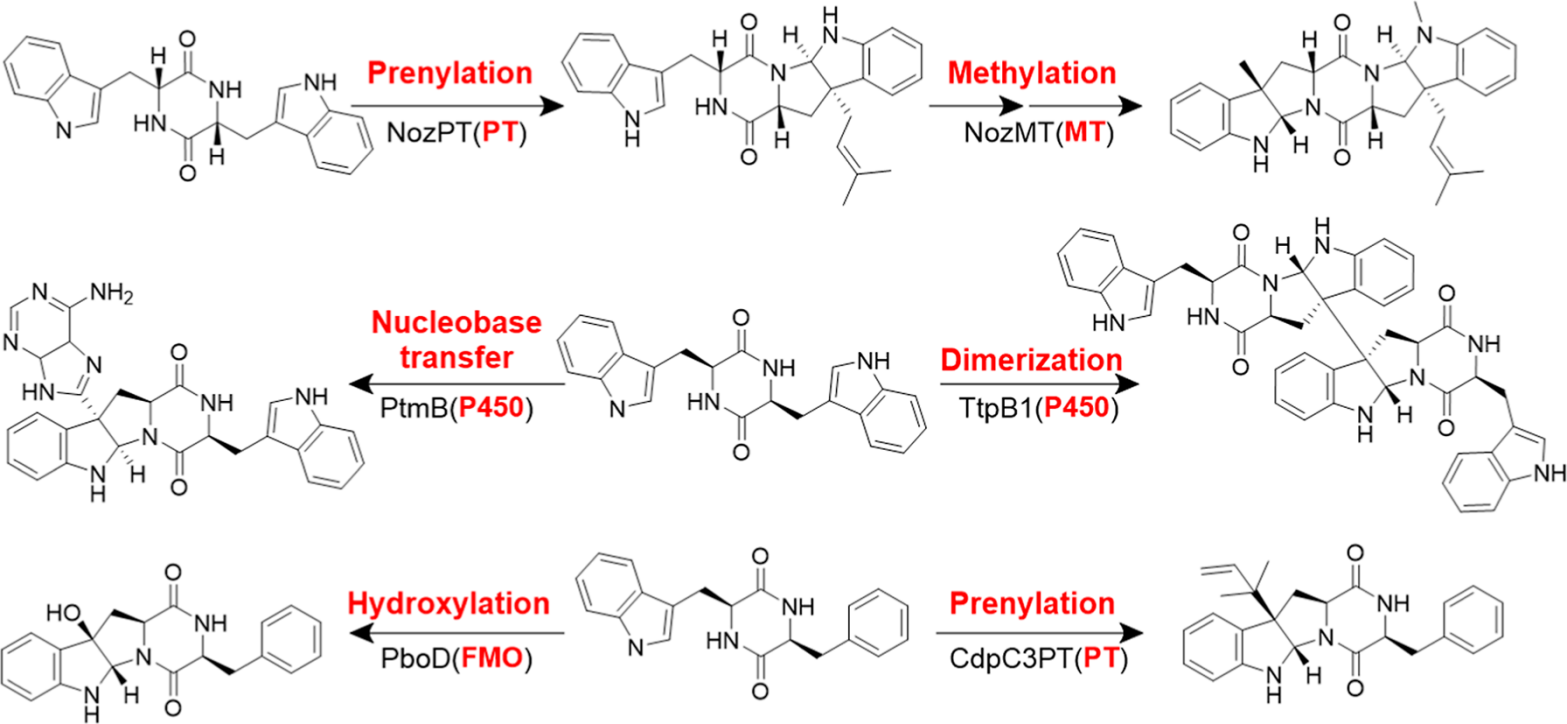
Representative diketopiperazine derivatives featuring the pyrrolo-[2,3-*b*]-indole scaffold and the enzymes for their formation.

## 2 Materials and Methods

### 2.1 General Experimental Procedures

Optical rotations were measured on a Jasco DIP-370 digital polarimeter (Jasco, Tokyo, Japan) using the sodium d-line (λ = 589 nm). Samples were dissolved in CHCl_3_ for analysis. NMR spectra were taken on a JEOL ECZ400S or ECA500 MHz spectrometer (JEOL, Akishima, Tokyo, Japan) and processed using MestReNov.14.2.1 (Mestrelab Research, Santiago de Compostella, Spain). Samples were prepared in CDCl_3_, CD_3_OD, or DMSO-*d*_6_. All chemical shifts are referred to those of the solvent signals. LC-MS analysis was conducted on an Agilent HPLC 1260 series system equipped with a Bruker microTOF QIII mass spectrometer. The separation was carried out with a VDSpher PUR100 C18-M-SE- column (3 μm, 150 × 2.0 mm, VDS optilab Chromatographie Technik) at a flow rate of 0.3 mL/min. The elution employed a linear gradient from 5 to 100% ACN in H_2_O, containing 0.1% (v/v) HCOOH, in 10 and 30 min were used. The column was then washed with 100% ACN for 5 min and equilibrated with 5% (*v/v*) ACN in H_2_O for 5 min, containing 0.1% (v/v) HCOOH. Data collection and analysis were performed with the Compass DataAnalysis 4.2 software (Bruker, Bremen, Germany).

Enzyme assays were analyzed on an Agilent HPLC 1260 with an Agilent Poroshell 120 EC-C18 column (2.7 μm, 100 × 3.0 mm). The substances were eluted at a flow rate of 0.3 mL/min with a linear gradient from 5 to 100% ACN in H_2_O, both containing 0.1% (v/v) HCOOH, in 20 min. The column was then washed and equilibrated as for the LC-MS analysis. Detection was carried out on a photodiode array detector.

Semipreparative HPLC was performed on the same equipment with a VDSpher PUR 100 C18-M-SE column (5 μm, 250 × 10 mm, VDS optilab Chromatographie Technik) and ACN/H_2_O without acid at a flow rate of 2 mL/min. Separation was done by stepped gradient elution.

Preparative HPLC was conducted using an Agilent 1260 Infinity II system with an Agilent Eclipse VDSpher PUR100C18-M-SE column (5 μm, 150 × 20 mm) at 15.0 mL/min, employing ACN in H_2_O as solvents.

### 2.2 Chemicals

Dimethylallyl diphosphate (DMAPP) was prepared according to the method described for geranyl diphosphate.^18^ Cyclodipeptides were prepared as described previously^19–21^ or purchased from Bachem (Bubendorf, Switzerland).

### 2.3 Strains, Media, and Growing Conditions

Strains used in this study are listed in Table S1. *Escherichia coli* DH5α and BL21­(DE3), *Saccharomyces cerevisiae* BJ5464-npgA, and *P. polonicum* strains were maintained and cultivated according to previously described methods.^22^

### 2.4 Extraction of Genomic DNA and RNA as Well as cDNA Synthesis

Mycelia of *P. polonicum* strains were cultivated in potato dextrose (PD) medium (24 g/L) for 2–3 days, collected, and transferred to 2 mL screw cap vials. Isolation of genomic DNA and RNA, as well as synthesis of complementary DNA, were performed as described previously.^23^

### 2.5 Gene Amplification, Gene Cloning, and Plasmid Construction

Plasmids generated and used in this study are listed in Table S2. The primers used for PCR amplification were synthesized by Seqlab GmbH (Göttingen, Germany) and are listed in Table S3. PCR amplification was performed with the Phusion High-Fidelity DNA polymerase from New England Biolabs (NEB) on a T100 Thermal Cycler (Bio-Rad). Plasmids for gene deletion and heterologous expression were constructed by homologous recombination in *E. coli* or *S. cerevisiae*.^24,25^

### 2.6 Genetic Manipulation and Cultivation of *P. polonicum*

Fungal germination, protoplast formation, and transformation were carried out according to the protocol described before.^22^ The potential transformants were verified via PCR amplification and cultivated on rice at 25 °C for 14 days. The EtOAc extracts were dissolved in methanol and analyzed by LC-MS.

Gene disruption was performed by homologous recombination. For sequential deletion of backbone genes, an auxotrophy-based selection system was employed using a *pyrG*-deficient strain. For *pyrG* deletion, about 1.5 kbp of the upstream and downstream flanking regions of *pyrG* were amplified and subsequently fused by PCR to generate a deletion cassette, which was then transformed into the wild-type strain to replace the *pyrG* locus. The resulting Δ*pyrG* strain was then used for auxotrophic selection, allowing *pyrG* to be reused as a recyclable marker for further gene deletions. Selection of Δ*pyrG* strains was carried out on medium at pH 4 supplemented with 1 mg/mL 5-fluoroorotic acid (5-FOA), 0.5 g/L uridine, and 0.5 g/L uracil. Targeted backbone gene disruption and marker recycling were performed using a split-marker strategy according to a previous protocol.^26^ The deletion cassette consisted of two fragments. One of them was generated by fusion of approximately 1.5 kbp upstream flanking region of the target gene and two-thirds of the *pyrG* marker from the 5′-end. The second fragment was amplified from a plasmid template constructed in *S. cerevisiae* via homologous recombination based on the NotI-linearized pYH-gpdA vector. This fragment contained 0.3 kbp of the upstream flanking sequence and approximately 1.5 kbp of the downstream of the target gene inserted behind the *pyrG* marker of pYH-*gpdA*, to enable marker recycling. Deletion of *vftPT* and *vftAT* was carried out using a split-marker strategy as described previously, with hygromycin B resistance (*hph*) as selection marker.^22^

### 2.7 Overproduction and Purification of VftPT and VftAT

For VftPT and VftAT overproduction, their coding regions were amplified from cDNA and cloned into pET28a­(+), resulting in the expression plasmids pHLW75 and pHLW77, respectively. These plasmids were then transferred into *E. coli* BL21­(DE3) and cultivated in Terrific Broth (TB) medium (2.4% yeast extract, 2.0% tryptone, 0.4% glycerol, 0.1 M phosphate buffer, pH 7.4). Overproduction of VftPT and VftAT was induced with 0.1 mM IPTG at 16 °C for 20 h. The His_6_-tagged recombinant proteins were purified by Ni-NTA affinity chromatography (Qiagen) to nearly homogeneity, which was confirmed on a sodium dodecyl sulfate-polyacrylamide gel (12%). For comparison, CdpC3PT^12^ and CdpNPT^27^ were heterologously overproduced in *E. coli* and purified as described previously.

### 2.8 Secondary Metabolite Extraction and Isolation

For structural elucidation of metabolites of interest, the fungal strains were cultivated on rice or PD media at 25 °C for 14 days. The cultures were treated with EtOAc and the extracts were purified by silica gel 60 (0.04–0.063 mm and 0.2–0.5 mm) column chromatography. Viridicatol (**2**), viridicatin (**3**), and 3-*O*-methylviridicatin (**4**) were isolated from the extract of *P. polonicum* NRRL995 by using semipreparative HPLC with acetonitrile/water (50:50, v/v) or (40:60, v/v) as elution solvents. Deoxyverrucosidin (**5**) was isolated as described previously.^22^

To isolate verrucofortine (**1**) from *P. polonicum* Δ*vodB2*Δ*dovA*, the crude extract was fractionated by preparative HPLC using a linear gradient from 5 to 100% ACN in H_2_O over 30 min, resulting in 4 fractions (1–4). Fraction 2 was further purified via semipreparative HPLC using a linear gradient from 30 to 75% ACN in H_2_O over 30 min, yielding verrucofortine (**1**).

*Cyclo*- *l* -Trp- *l* -Leu (**6**) was isolated from a PD culture of *P. polonicum* Δ*vdoB2*Δ*dovA*Δ*vftPT*. The crude extract was fractionated by preparative HPLC using a linear gradient of 5–100% ACN in H_2_O over 30 min to afford three fractions. Fraction 2 was further purified by semipreparative HPLC with a linear gradient of 20–55% ACN in H_2_O over 20 min, yielding compound **6**. *Allo*-brevicompanine B (**7**) was isolated from a PD culture of *P. polonicum* Δ*vdoB2*Δ*dovA*Δ*vftAT* under the same preparative HPLC conditions as for **6**, with final purification by semipreparative HPLC (50–80% ACN in H_2_O over 30 min).

To isolate the enzymatic products **7a**–**7g**, the incubation mixtures of VftPT were extracted with EtOAc twice. The extracts were purified via semipreparative HPLC using a linear gradient of 5–100% ACN in H_2_O containing 0.1% (v/v) HCOOH over 20 min, affording compounds **7a**–**7g**.

### 2.9 In Vitro Assays with Recombinant Proteins

Standard prenyltransferase assays in 50 μL contained 50 mM Tris–HCl (pH 7.5), 5 mM CaCl_2_, 1 mM cyclodipeptides (**6** and **6a**–**6g**), 2 mM DMAPP, and 1 μM of one of the purified recombinant enzymes VftPT, CdpC3PT, or CdpNPT. To test the enzyme activity of VftAT, reaction mixtures (50 μL) contained 50 mM Tris–HCl (pH 7.5), 1 mM *allo*-brevicompanine B (**7**), 1 mM acetyl-CoA, and 0.4 μM purified VftAT. The mixtures were incubated at 37 °C for 30 min and terminated by addition of an equal volume of methanol. Samples were centrifuged (13,000*g*, 20 min) and analyzed on LC-MS.

Enzyme assays for kinetic analysis were performed in 50 μL reaction mixtures containing 50 mM Tris–HCl (pH 7.5), 5 mM CaCl_2_, and purified recombinant proteins. Kinetic parameters of VftPT for DMAPP were determined by using 0.07 μM enzyme, 2 mM *cyclo*- *l* -Trp- *l* -Leu (**6**), and DMAPP at concentrations of 0–5.0 mM. For determination of its kinetic parameters toward cyclodipeptides, DMAPP was fixed at 2 mM. 0.3 μM CdpC3PT and 0.5 μM CdpNPT were used respectively for determination of their kinetic parameters toward cyclodipeptides (**6** and **6a**–**6g**) in the presence of 5 mM DMAPP. Due to its low solubility, the concentration of *cyclo*- *l* -Trp- *l* -Tyr was limited to 1.0 mM, whereas final concentrations of up to 5.0 mM were used for other cyclodipeptides.

To determine the kinetic parameters of VftAT toward acetyl-CoA, 2 mM *allo*-brevicompanine B (**7**) was used as the substrate, with final acetyl-CoA concentrations of up to 5.0 mM. For assessing the kinetic parameters of VftAT toward *allo*-brevicompanine B (**7**), acetyl-CoA was set at 2 mM. The reaction mixtures in 50 μL contained 50 mM Tris–HCl (pH 7.5), 0.04 μM of purified recombinant VftAT and **7** with final concentration of up to 5.0 mM.

All reaction mixtures were incubated at 37 °C for 30 min and terminated by addition of 50 μL methanol. Proteins were removed by centrifugation at 13,000*g* for 20 min. The assays were carried out in three independent replicates. SEMs are given as ± values. Kinetic parameters were calculated by nonlinear regression of the Michaelis–Menten equation using GraphPad Prism 10.0.

### 2.10 Physicochemical Properties of the Compounds Described in This Study

**Verrucofortine** (**1**) pale yellow solid; [α]_*D*_^20^ = −170.6°(c 0.2, CHCl_3_); ^1^H NMR data (Table S6) and spectrum (Figure S9); HRESIMS *m*/*z*: 410.2456 [M + H]^+^, calcd for C_24_H_32_N_3_O_3_, 410.2438 (Figure S24).

**Viridicatol** (**2**) yellow solid; ^1^H NMR data (Table S7) and spectrum (Figure S10); HRESIMS *m*/*z*: 254.0806 [M + H]^+^, calcd for C_15_H_12_NO_3_, 254.0812 (Figure S24).

**Viridicatin** (**3**) yellow solid; ^1^H NMR data (Table S7) and spectrum (Figure S11); HRESIMS *m*/*z*: 238.0861 [M + H]^+^, calcd for C_15_H_12_NO_2_, 238.0863 (Figure S24).

**3-*O*-methylviridicatin** (**4**) yellow solid; ^1^H NMR data (Table S7) and spectrum (Figure S12); HRESIMS *m*/*z*: 252.1014 [M + H]^+^, calcd for C_16_H_14_NO_2_, 252.1019 (Figure S24).

***Cyclo*-** ***l*** **-Trp-** ***l*** **-Leu** (**6**) white solid; ^1^H NMR data (Table S8) and spectrum (Figure S13); HRESIMS *m*/*z*: 300.1722 [M + H]^+^, calcd for C_17_H_22_N_3_O_2_, 300.1707 (Figure S24).

***Allo*-brevicompanine B** (**7**) white solid; [α]_*D*_^20^ = −265°(c 0.2, CHCl_3_); ^1^H-data (Table S8) and spectrum (Figure S14); HRESIMS *m*/*z*: 368.2321 [M + H]^+^, calcd for C_22_H_30_N_3_O_2_, 368.2333 (Figure S24).

**7a**: white solid; ^1^H NMR data (Table S9) and spectrum (Figure S15); HRESIMS *m*/*z*: 418.2115 [M + H]^+^, calcd for C_25_H_28_N_3_O_3_, 418.2125 (Figure S25).

**7b**: white solid; ^1^H NMR data (Table S9) and spectrum (Figure S16); HRESIMS *m*/*z*: 402.2174 [M + H]^+^, calcd for C_25_H_28_N_3_O_2_, 402.2176 (Figure S25).

**7c**: white solid; ^1^H NMR data (Table S10) and spectrum (Figure S17); HRESIMS *m*/*z*: 441.2287 [M + H]^+^, calcd for C_27_H_29_N_4_O_2_, 441.2285 (Figure S25).

**7d**: white solid; ^1^H NMR data (Table S10) and spectrum (Figure S18); HRESIMS *m*/*z*: 352.2011 [M + H]^+^, calcd for C_21_H_26_N_3_O_2_, 352.2020 (Figure S25).

**7e**: white solid; ^1^H NMR data (Table S11) and spectrum (Figure S19); HRESIMS *m*/*z*: 326.1867 [M + H]^+^, calcd for C_19_H_24_N_3_O_2_, 326.1863 (Figure S25).

**7f**: white solid; ^1^H NMR data (Table S11) and spectrum (Figure S20); HRESIMS *m*/*z*: 392.2074 [M + H]^+^, calcd for C_22_H_26_N_5_O_2_, 392.2081 (Figure S25).

**7g**: white solid; ^1^H NMR data (Table S12) and spectrum (Figure S21); HRESIMS *m*/*z*: 312.1701 [M + H]^+^, calcd for C_18_H_22_N_3_O_2_, 312.1707 (Figure S25).

## 3 Results and Discussion

### 3.1 Genome Mining and Identification of the Verrucofortine Biosynthetic Gene Cluster

Given the limited information on the biosynthesis of verrucofortine, we intended to identify its biosynthetic gene cluster (BGC) in fungi. Mining of the *P. polonicum* NRRL995 genome by using the antiSMASH 7.0 platform^28^ led to predict 61 BGCs. Among them, one distinct locus, designed as *vft* cluster (Table S4 and Figure 2A), would meet the requirement for the biosynthesis of verrucofortine. It consists of three genes coding for a nonribosomal peptide synthetase VftPS, a prenyltransferase VftPT, and an acetyltransferase VftAT. The VftPS protein with a canonical bimodular NRPS architecture A-T-C-A-T-C shares a sequence identity of 52% with PboA, an enzyme for the formation of *cyclo*- *l* -Trp- *l* -Leu by condensation of *l* -tryptophan and *l* -leucine.^29^ VftPT shows significant sequence identifies to known cyclodipeptide prenyltransferases. Phylogenetic analysis revealed that VftPT is located closely to enzymes such as RoqD,^30^ CdpC3PT,^12^ and CdpNPT^27^ for *C3ß*-prenylation of tryptophan-containing cyclodipeptides at the indole ring (Figure S1). These bioinformatic clues collectively support the *vft* cluster as an interesting candidate for the biosynthesis of verrucofortine.

**Figure 2:**
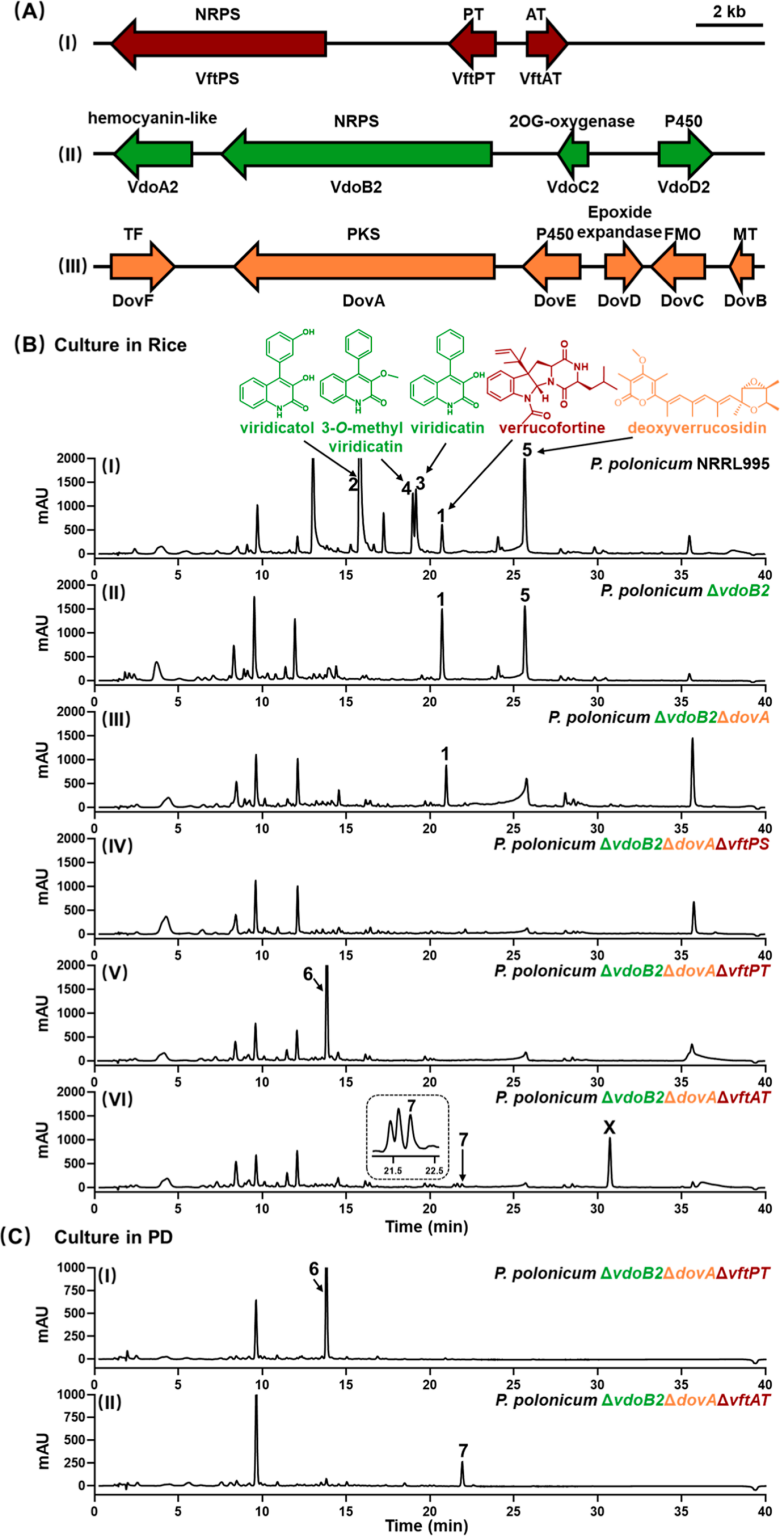
The *vft*, *vdo2*, and *dov* gene clusters in *P. polonicum* and their responsibility for the metabolite biosynthesis. (A) Schematic illustration of the clusters; (B) HPLC chromatograms of 14 day-old rice cultures; (C) HPLC chromatograms of 14 day-old PD cultures. The metabolites were detected on a photo array detector and illustrated for UV absorption at 280 nm.

### 3.2 Construction of a Low-Metabolite-Background Strain to Facilitate Product Detection

*P. polonicum* NRRL995 produces a number of secondary metabolites (Figure 2B) including viridicatol (**2**), viridicatin (**3**), and 3-*O*-methylviridicatin (**4**), which were isolated and identified in this study (Table S7 and Figures S10–S12),^31,32^ as well as deoxyverrucosidin and analogues, recently investigated by our group.^22^ One minor peak with a retention time at 21 min and a [M + H] ^+^ ion at *m*/*z* 410.2456 shows maximal UV absorptions at 247, 277, and 285 nm, corresponding to those of verrucofortine.^8^ To reduce the number of highly accumulated products and to provide a strain with low-metabolite-background, we first constructed a *P. polonicum* strain by deletion of their backbone genes. For this purpose, the frequently used recyclable selection marker *pyrG* gene, coding for an orotidine-5′-phosphate decarboxylase^26^ was first deleted via homologous recombination and selection with uracil/uridine auxotrophy in the presence of 5-FOA. PCR verification confirmed the precise deletion of the *pyrG* locus, yielding a strain suitable for subsequent iterative genome editing (Figure S2).

With the recyclable *pyrG* marker established, we next targeted the BGCs responsible for the viridicatol and deoxyverrucosidin biosynthesis. Comparative genomic analysis revealed the presence of a candidate BGC, termed *vdo2* (Table S4), for the biosynthesis of viridicatin-type metabolites with more than 90% sequence identity to those of the previously characterized *vdo* cluster from *Penicillium palitans* for viridicatol biosynthesis (Figures 2AII and S3).^33^ No putative methyltransferase gene was predicted in the *vdo2* cluster. It is likely that the conversion of viridicatin (**3**) to 3-*O*-methylviridicatin (**4**) is catalyzed by a promiscuous enzyme encoded by a gene outside the *vft* cluster. We sequentially deleted the backbone genes for the formation of viridicatol and deoxyverrucosidin, *i*.*e*. the NRPS gene *vdoB2* and the PKS gene *dovA* (Figures 2AII and 2AIII), in the Δ*pyrG* strain. After verification by PCR (Figure S2), the deletion mutants were cultivated and the metabolite changes were monitored on LC-MS. As shown in Figure 2B, deletion of these two genes led to complete abolishment of the production for viridicatin- and deoxyverrucosidin-type metabolites (Figures 2BI–III), providing a strain for convenient genetic manipulation.

### 3.3 Identification of Verrucofortine and Its Biosynthetic Gene Cluster

To confirm the involvement of the *vft* cluster in the biosynthesis of a prenylated and acetylated cyclodipeptide like verrucofortine, the NRPS gene *vftPS* was deleted in the constructed strain *P. polonicum* Δ*vodB2*Δ*dovA* (Figures 2AI and S2). LC-MS analysis of the Δ*vftPS* mutant revealed disappearance of the peak at 21 min mentioned above (Figures 2BIII and 2BIV), proving the involvement of the *vft* cluster for its formation. To elucidate the structure of the peak at 21 min, the strain Δ*vdoB2*Δ*dovA* without deoxyverrucosidin accumulation was used for cultivation in rice culture, which is easier for purification, despite slightly lower yield for the peak of interest was detected than in the Δ*vdoB2* strain. After isolation on HPLC, the structure is confirmed by NMR and MS analyses to be verrucofortine, an *N1*-acetylated and *C3ß*-prenylated pyrrolo-[2,3-*b*]-indole derivative originated from *cyclo*- *l* -Trp- *l* -Leu (Table S6 and Figures 2B and S9). This structure meets perfectly the genetic information on the *vft* cluster illustrated in Figure 2AI. Based on the sequence homology of the genes from the *vft* cluster with their homologues (Table S4), we proposed a biosynthetic pathway for verrucofortine in *P. polonicum* (Figure 3A). The NRPS VftPS assembles the cyclodipeptide **6** by condensation of l-Trp and l-Leu, which is then modified by prenylation with VftPT and acetylation with VftAT. Based on the biosynthesis of known *C3*-prenylated fungal pyrrolo-[2,3-*b*]-indole derivatives like aszonalenin^34^ and roquefortine C/meleagrin,^30,35^ it can be proposed that the VftPT reaction takes place before that of VftAT, which should be proved by gene deletion and biochemical investigations.

**Figure 3:**
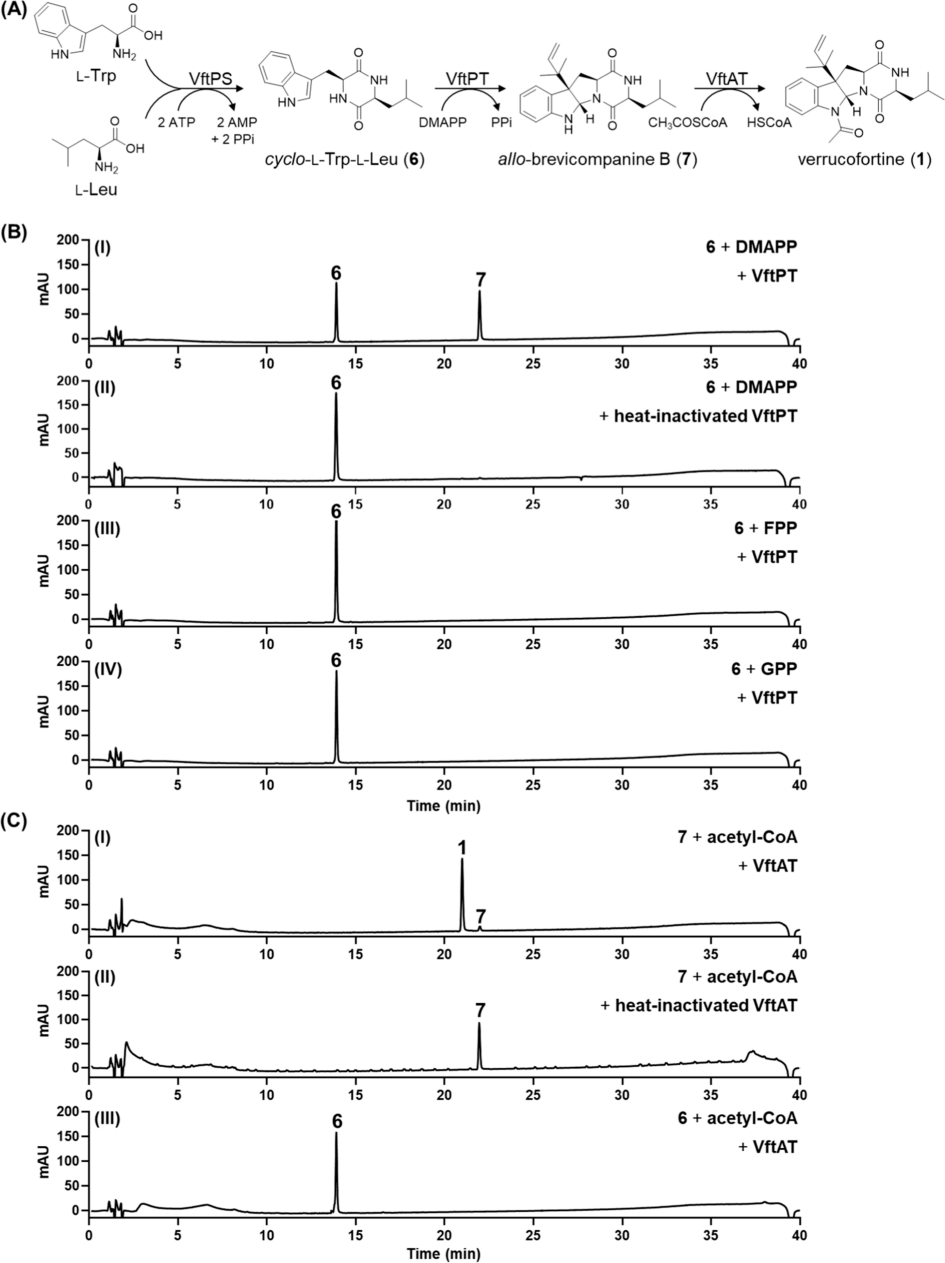
Enzymes involved in the biosynthesis of verrucofortine (**1**). (A) Proposed reaction scheme; (B) HPLC chromatograms of enzyme assays of VftPT with **6**; (C) HPLC chromatograms of enzyme assays of VftAT with **7** and **6**. The chromatograms were illustrated for UV absorptions at 254 nm.

Targeted gene deletion experiments were afterward conducted on *vftPT* and *vftAT*. Deletion of *vftPT* and *vftAT* completely abolished verrucofortine production and led to accumulation of one product peak each in the resulted mutants, *i.e.,* **6** in Δ*vftPT* and **7** in Δ*vftAT* (Figures 2BV and 2BVI). Interestingly, similar product yields for **6** in Δ*vftPT* were observed in both rice and PD media (Figures 2CI). In contrast, much higher product formation for **7** in Δ*vftAT* was detected in PD than in rice culture (Figures 2CII). Instead, a significant peak X at 31 min with a [M + H]^+^ ion at *m*/*z* 614.3853 was accumulated in rice culture, indicating the increased production of a product to be characterized in a future study.

Isolation of **6** and **7** from PD cultures and structural elucidation confirmed them to be the NRPS product *cyclo*- *l* -Trp- *l* -Leu and the deacetylated verrucofortine, *allo*-brevicompanine B, respectively. *Allo*-brevicompanine B was isolated from a deep ocean sediment-derived *Penicillium* strain.^36^ These results provide direct evidence for the proposed pathway in Figure 3A.

### 3.4 Biochemical Characterization of VftPT and VftAT

To characterize these enzymes biochemically, VftPT and VftAT were heterologously overproduced in *E. coli* and purified to near homogeneity. SDS-PAGE analysis confirmed the expected size and high purity (Figure S4).

The activity of VftPT was examined by using **6** as a prenyl acceptor and DMAPP as the donor. As shown in Figure 3BI, **6** was clearly converted by VftPT to its *C3ß*-prenylated derivative, *allo*-brevicompanine B (**7**), as confirmed by LC-MS and NMR analyses (Table S8 and Figure S14). In comparison, no product was detected in the control with heat-inactivated enzyme (Figures 3BI and 3BII). These results prove that VftPT functions as a cyclodipeptide prenyltransferase catalyzing the *C3ß*-prenylation at the indole residue with simultaneous ring formation between the original indole diketopiperazine to afford pyrrolo-[2,3-*b*]-indole with a 6/5/5/6 fused ring system. Prenyltransferases are generally proposed to catalyze prenylation via an electrophilic mechanism.^34,37,38^ In the case of *C3*-prenylation, the formed cation at C2 of the indole ring after attachment of the prenyl moiety at C3 is attacked by the electron-rich nitrogen at the diketopiperazine ring. The pyrrolo-[2,3-*b*]-indole scaffold is then formed by elimination of one proton from this nitrogen.^34^ In comparison, the prenylations with retention of the indole ring, e.g. at N1, C2, and C4–C7, is an electrophilic substitution of one proton at these positions.^39,40^

Based on the crystal structures of some prenyltransferases like FgaPT2,^37^ FtmPT1,^38^ and CdpNPT,^40^ several key residues in the active sites, corresponding to T102, K174, R244, and Y191 in FgaPT2, were identified. Mutagenetic studies at these positions revealed their responsibility for substrate recognition, prenylation position and pattern.^38,41,42^ Sequence alignments of VftPT with FgaPT2 and serval cyclodipeptide *C2*- and *C3*-prenyltransferases show conservation and differences at these positions, even within the *C3ß*-prenyltransferases VftPT, CdpNPT and CdpC3PT (Figure S5). It is likely that several residues in the active site determine together the catalytic behavior of the respective prenyltransferase.

To evaluate its substrate specificity toward donors, DMAPP was replaced by farnesyl diphosphate (FPP) or geranyl diphosphate (GPP). VftPT failed to utilize these substrates, indicating its high donor specificity (Figures 3BIII and 3BIV).

VftAT was assayed afterward by using the prenylated intermediate (**7**) as substrate in the presence of acetyl-CoA, leading to detection of the acetylated product verrucofortine (**1**), while no product formation was detected in control assay with heat-inactivated enzyme (Figures 3CI and 3CII). In addition, no product was observed in the assay of **6** and acetyl-CoA with VftAT (Figure 3CIII), proving VftPT as the first and VftAT the last tailoring enzyme in the proposed pathway depicted in Figure 3A.

### 3.5 Kinetic Parameters of VftPT and VftAT toward Their Natural Substrates

Kinetic parameters of VftAT and VftPT were determined to assess their catalytic efficiencies. As shown in Figure 4, both VftPT and VftAT reactions follow the Michael–Menten kinetics. VftPT displayed high affinity and catalytic activities to both substrates with comparable *K*_M_ values of approximate 0.1 mM and turnover number 1.3/1.4 s^–1^. The catalytic efficiency *k*_cat_/*K*_M_ was calculated for DMAPP at 13,200 s^–1^ M^–1^ and *cyclo*- *l* -Trp- *l* -Leu (**6**) at 10,846 s^–1^ M^–1^ (Figure 4A,B). In contrast, VftAT exhibited much higher affinity to its acetyl donor than acceptor with *K*_M_ value of 0.1 mM for acetyl-CoA and 0.54 mM for *allo*-brevicompanine B (**7**). Very high turnover numbers (7.61/8.30 s^–1^) were calculated for both substrates (Figure 4C,D). The much higher catalytic efficiency of VftAT than VftPT could be the reason for the absence of *allo*-brevicompanine B (**7**) in the strain NRRL995 (Figure 2BI).

**Figure 4:**
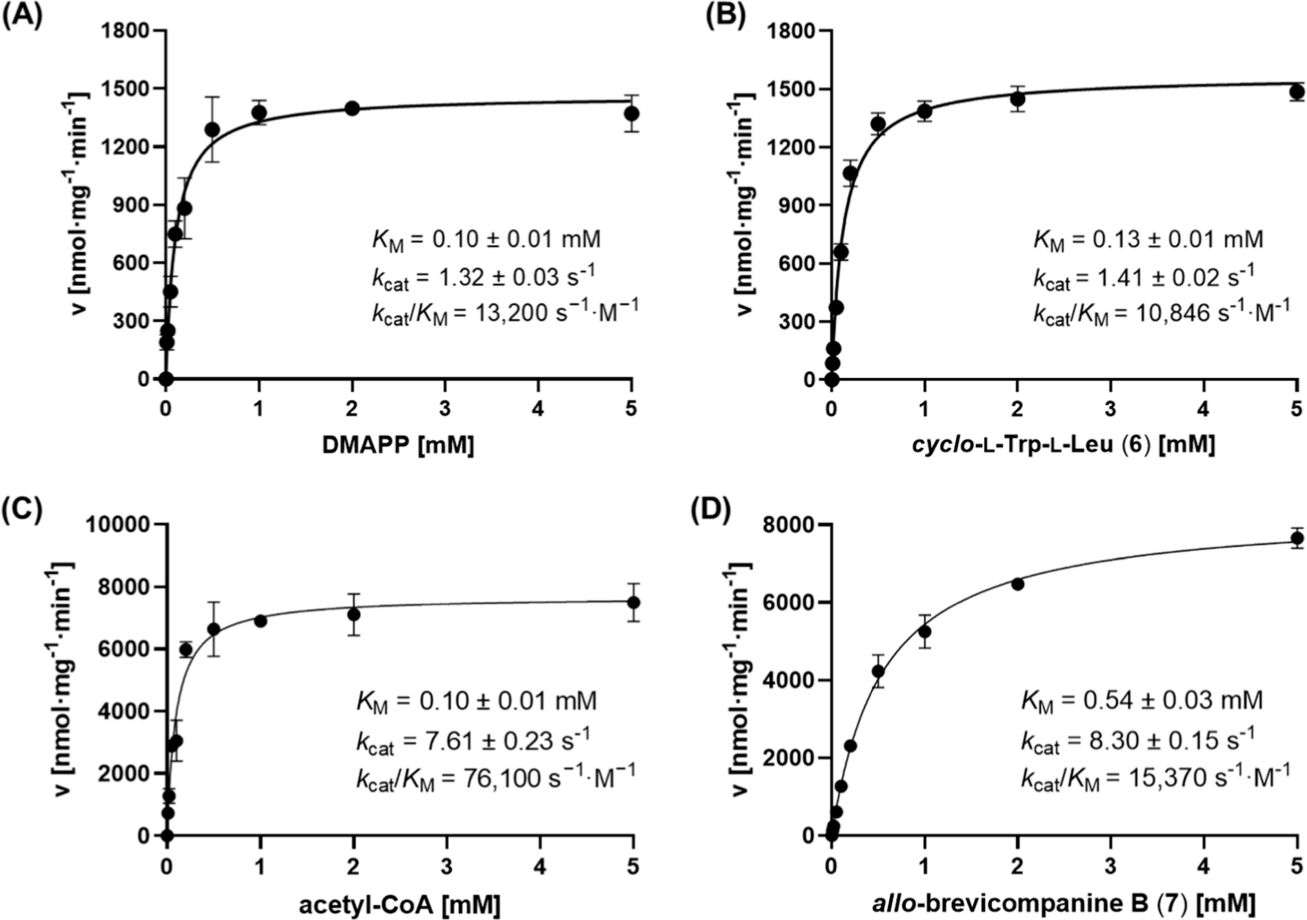
Kinetic parameters of VftPT and VftAT toward their natural substrates. (A,B) VftPT toward DMAPP and *cyclo*- *l* -Trp- *l* -Leu, (C,D) VftAT toward acetyl-CoA and *allo*-brevicompanine B.

### 3.6 Substrate Specificity and Catalytic Efficiency of VftPT Compared to Its Homologues

Phylogenetic analysis identified CdpC3PT and CdpNPT as the two enzymes most closely related to VftPT (Figure S1). To assess their substrate specificity and catalytic performance, the three enzymes were incubated parallelly with eight tryptophan-containing cyclodipeptides in the presence of DMAPP with same enzyme concentration under identical reaction conditions. These cyclodipeptides include *cyclo*- *l* -Trp- *l* -Leu (cWL, **6**), *cyclo*- *l* -Trp- *l* -Tyr (cWY, **6a**), *cyclo*- *l* -Trp- *l* -Phe (cWF, **6b**), *cyclo*- *l* -Trp- *l* -Trp (cWW, **6c**), *cyclo*- *l* -Trp- *l* -Pro (cWP, **6d**), *cyclo*- *l* -Trp- *l* -Ala (cWA, **6e**), *cyclo*- *l* -Trp- *l* -His (cWH, **6f**), and *cyclo*- *l* -Trp-Gly (cWG, **6g**), representing diverse physicochemical features in side-chain size, hydrophobicity, and aromaticity. Comparison of retention times, UV spectra, MS, and MS^2^ as well as NMR analysis after isolation from the VftPT assays confirmed **7** and **7a**–**7g** with the same structures as the products of CdpC3PT and CdpNPT for a given cyclodipeptide (Tables S8–S12 and Figures S14–S21).

Obviously, VftPT consistently exhibited higher activity than both CdpC3PT and CdpNPT toward all tested cyclodipeptides with product yields of 33.4–85.7%, regardless of whether they are natural or non-natural substrates (Figure 5). In contract, CdpC3PT showed higher activity only toward cWL and cWP with product yields of more than 30%, while conversions of other cyclodipeptides remained lower than 15%. A similar substrate preference was also observed for CdpNPT, which afforded product yields of 32.9% for cWP and 17.9% for cWW, whereas conversions for the remaining cyclodipeptides are less than 10.5%.

**Figure 5:**
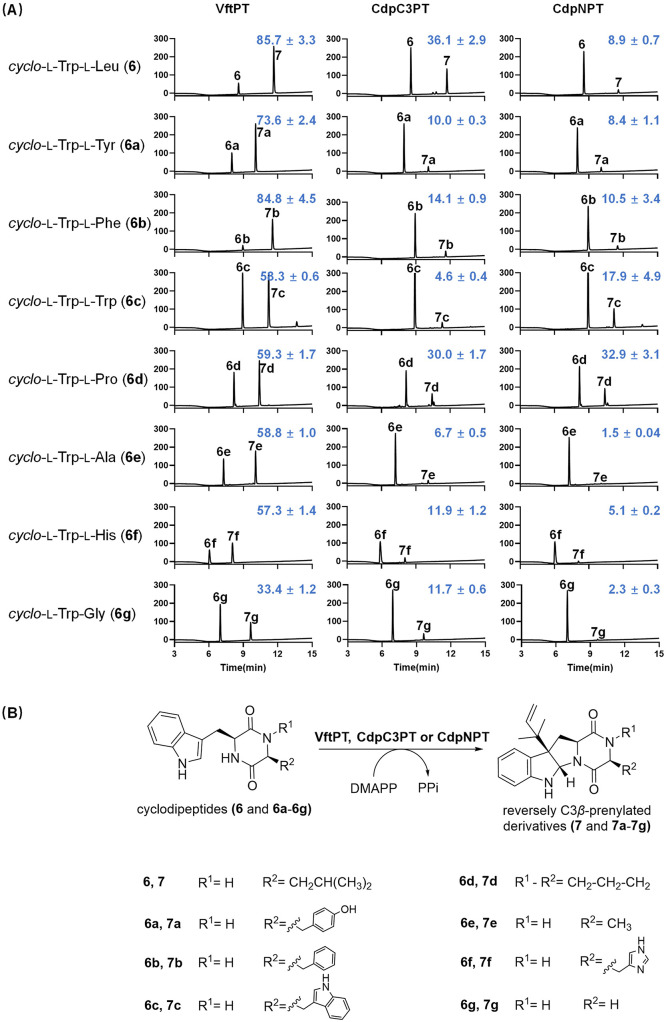
Conversion of selected cyclodipeptides (**6** and **6a**–**6g**) by VftPT, CdpC3PT, and CdpNPT. (A) HPLC chromatograms of the incubation mixtures were monitored for absorptions at UV 254 nm. The conversion rates to the products **7** and **7a**–**7g** (%) are given in blue at the upper right corner of each panel; (B) prenylations of **6** and **6a**–**6g** by VftPT, CdpC3PT, and CdpNPT in the presence of DMAPP.

The kinetic parameters of VftPT, CdpC3PT, and CdpNPT were then determined to further evaluate their preference toward these cyclodipeptides and to allow direct comparison (Table 1 and Figures S6–S8). All three enzymes exhibited typical Michaelis–Menten kinetics, with the reaction velocity increasing in a substrate concentration-dependent manner (Figures S6–S8). The *k*_cat_/*K*_M_ values were generally consistent with the observed conversion rates in Figure 5, confirming that VftPT displays higher catalytic efficiency toward the tested substrates relative to its close homologues CdpC3PT and CdpNPT. As summarized in Figure S6, VftPT exhibited *K*_M_ values of 0.14–0.23 mM for **6a**–**6g**, being comparable or slightly higher than that of **6** at 0.10 mM. The corresponding turnover numbers (*k*_*cat*_) ranged from 1.29 ± 0.02 s^–1^ for **6a** to 0.24 ± 0.01 s^–1^ for **6g**. Consistently, VftPT displayed the highest catalytic efficiencies across all tested substrates, reaching a maximum *k*_cat_/*K*_M_ of 10,846 s^–1^ M^–1^ for cWL and decreasing to 1263 s^–1^ M^–1^ for cWG, which is roughly 1 order of magnitude higher than either CdpC3PT or CdpNPT (Table 1). In contrast, CdpC3PT and CdpNPT showed generally comparable low activities in the range of 125–583 s^–1^ M^–1^ and 143–833 s^–1^ M^–1^, respectively. CdpC3PT displayed slightly higher activity toward cWL (583 s^–1^ M^–1^), whereas CdpNPT reached its maximum for cWP (833 s^–1^ M^–1^).

**Table 1:** Comparison of Catalytic Efficiencies (s^–1^ M^–1^) of VftPT with CdpC3PT and CdpNPT

	VftPT	CdpC3PT	CdpNPT
**6**	10,846	583	309
**6a**	9214	206	285
**6b**	8267	282	357
**6c**	6667	125	417
**6d**	6438	556	833
**6e**	5200	159	192
**6f**	4348	208	231
**6g**	1263	200	143

Compared with other reported cyclodipeptide prenyltransferases listed in Table S5, such as BrePT, CdpC2PT, and CdpC7PT, which show high activity toward their natural substrates, but very low activity toward other cyclodipeptides. FtmPT1 exhibits high activity for five cyclodipeptides mentioned in this study, but shows significant prefer toward its natural substrate **6e**, at least four-fold higher than for other tested cyclodipeptides. In comparison, VftPT maintains high and more balanced activity across diverse cyclodipeptides, emphasizing its potential for chemoenzymatic synthesis and synthetic biology of diverse cyclodipeptides.

In conclusion, we identified and elucidated the biosynthetic pathway of verrucofortine in *P. polonicum* through a combination of bioinformatic, genetic and biochemical approaches. A low-metabolic-background strain enabled precise identification of the NRPS (VftPS) for the backbone formation, followed by functional characterization of the associated prenyltransferase (VftPT) and acetyltransferase (VftAT) as two tailoring enzymes. Kinetic analysis demonstrated that both VftPT and VftAT exhibit high affinity and turnover number for their natural substrates and VftPT shows a broad substrate promiscuity and higher conversion efficiency than its homologues like CdpC3PT and CdpNPT. These findings provide a comprehensive understanding of verrucofortine biosynthesis, highlight the modularity and efficiency of fungal cyclodipeptide tailoring enzymes, and establish a basis for using these enzymes to generate structurally diverse and biologically active prenylated cyclodipeptides.

## Supplementary Material



## Notes

Primers, plasmids, and strains, NMR tables and spectra, UV and Mass spectra (PDF)

## Abbreviations Used

NRPS: nonribosomal peptide synthetase
PT: prenyltransferase
AT: acetyltransferase
DMAPP: dimethylallyl diphosphate
5-FOA: 5-fluoroorotic acid
FPP: farnesyl diphosphate
GPP: geranyl diphosphate
