# Supplementary material for: Elucidation of the Verrucofortine Biosynthetic Pathway Enables Identification of a Cyclodipeptide Prenyltransferase with High Catalytic Efficiency
Source: J Agric Food Chem. 2026 Mar 3;74(9):7752–62. doi: 10.1021/acs.jafc.6c01966 (PMC12983300; doi:10.1021/acs.jafc.6c01966)
Supplement: Supplementary file 1 [file jf6c01966_si_001.pdf]

Supporting Information

Elucidation of the Verrucofortine Biosynthetic Pathway Enables Identification of a Cyclodipeptide Prenyltransferase with High Catalytic Efficiency

Hui-Ling Wei,† Li-Dan Pang,† Xiao-Ling Chen, and Shu-Ming Li*

Philipps-Universität Marburg, Fachbereich Pharmazie, Institut für Pharmazeutische Biologie und Biotechnologie, Robert-Koch-Straße 4, 35037 Marburg, Germany

†These authors contributed equally

*Corresponding author. Email: shuming.li@staff.uni-marburg.de

Table of Contents

Supplementary Tables 4

Table S1. Strains constructed and used in this study 4

Table S2. Plasmids constructed and used in this study. 5

Table S3. Primers used in this study. 6

Table S4. Proteins encoded by the *vft* and *vdo2* cluster in *Penicillium polonicum* NRRL995. 9

Table S5. Catalytic efficiency (k_{cat}/K_M ($s^{-1}\cdot M^{-1}$)) of other reported PTs. 10

Table S6. The ^{1}H NMR (500 MHz) data of verrucofortine (**1**) in $CDCl_3$. 11

Table S7. The ^{1}H NMR (500 MHz) data of viridicatol (**2**), viridicatin (**3**) and 3-*O*-methylviridicatin (**4**) in DMSO-d_6. 12

Table S8. The ^{1}H NMR (400 MHz) data of *cyclo*-L-Trp-L-Leu (**6**) and *allo*-brevicompanine B (**7**) in $CDCl_3$. 13

Table S9. The ^{1}H NMR (400 MHz) data of **7a** and **7b** in $CDCl_3$. 14

Table S10. The ^{1}H NMR (400 MHz) data of **7c** and **7d** in $CDCl_3$. 15

Table S11. The ^{1}H NMR (400 MHz) data of **7e** in $CDCl_3$ and **7f** in CD_3OD. 16

Table S12. The ^{1}H NMR (400 MHz) data of **7g** in $CDCl_3$. 17

Supplementary Figures 18

Figure S1. Phylogenetic tree of prenyltransferases. 18

Figure S2. Target gene deletion and PCR verification for *P. polonicum* strain. 19

Figure S3. Comparison of viridicatol biosynthetic gene clusters in *P. palitans* and *P. polonicum*. 20

Figure S4. SDS-PAGE of the purified VftAT, VftPT, CdpC3PT, and CdpNPT. 21

Figure S5. Sequence alignments of FgaPT2, FtmPT1, NotF, BrePT, CdpC2PT, AnaPT, CdpC3PT, CdpNPT, and VftPT. 22

Figure S6. Kinetic parameters of VftPT toward selected cyclodipeptides (**6a**–**6g**). 23

Figure S7. Kinetic parameters of CdpC3PT toward selected cyclodipeptides (**6** and **6a**–**6g**). 24

Figure S8. Kinetic parameters of CdpNPT toward selected cyclodipeptides (**6** and **6a**–**6g**). 25

Figure S9. ^{1}H NMR spectrum of verrucofortine (**1**) in $CDCl_3$ (500 MHz). 26

Figure S10. ^{1}H NMR spectrum of viridicatol (**2**) in DMSO-d_6 (500 MHz). 27

Figure S11. ^{1}H NMR spectrum of viridicatin (**3**) in DMSO-d_6 (500 MHz). 28

Figure S12. ^{1}H NMR spectrum of 3-*O*-methylviridicatin (**4**) in DMSO-d_6 (500 MHz). 29

Figure S13. ^{1}H NMR spectrum of *cyclo*-L-Trp-L-Leu (**6**) in $CDCl_3$ (400 MHz). 30

Figure S14. ^{1}H NMR spectrum of *allo*-brevicompanine B (**7**) in $CDCl_3$ (400 MHz). 31

Figure S15. ^{1}H NMR spectrum of **7a** in $CDCl_3$ (400 MHz). 32

Figure S16. ^{1}H NMR spectrum of **7b** in $CDCl_3$ (400 MHz). 33

Figure S17. ^{1}H NMR spectrum of **7c** in $CDCl_3$ (400 MHz). 34

Figure S18. ^{1}H NMR spectrum of **7d** in $CDCl_3$ (400 MHz). 35

Figure S19. ^{1}H NMR spectrum of **7e** in $CDCl_3$ (400 MHz). 36

Figure S20. ^{1}H NMR spectrum of **7f** in CD_3OD (400 MHz). 37

Figure S21. ^{1}H NMR spectrum of **7g** in $CDCl_3$ (400 MHz). 38

Figure S22. UV spectra of verrucofortine (**1**), viridicatol (**2**), viridicatin (**3**), 3-*O*-methylviridicatin (**4**), *cyclo*-L-Trp-L-Leu (**6**), and *allo*-brevicompanine B (**7**). 39

Figure S23. UV spectra of **7a**–**7g**. 40

Figure S24. Mass spectra of verrucofortine (**1**), viridicatol (**2**), viridicatin (**3**), 3-*O*-methylviridicatin (**4**), *cyclo*-L-Trp-L-Leu (**6**), and *allo*-brevicompanine B (**7**). 41

Figure S25. Mass spectra of **7a**–**7g**. 42

References **43**

Supplementary Tables

Table S1. Strains constructed and used in this study.

Strain	Genotype	Source
E.coli		
DH5α	F⁻*endA1 glnV44 thi-1 recA1 relA1 gyrA96 deoR nupG purB20* φ80dl*acZ*ΔM15 Δ(*lacZYA-argF*)U169, hsdR17($r_K^-m_K^+$), λ⁻	[1]
BL21(DE3)	*fhuA2 [lon] ompT gal (λ DE3) [dcm] ΔhsdS* *λ DE3 = λ sBamHIo ΔEcoRI-B int::(lacI::PlacUV5::T7 gene1) i21 Δnin5*	[2]
S. cerevisiae		
BJ5464-npgA	*MATα ura3-52 leu2-Δ1 trp1 his3-Δ200 pep4::HIS3 prb1-Δ1.6R can1 GAL npgA*	[3]
Penicillium polonicum		
NRRL995	wild type	NRRL*
HLW109	Δ*pyrG* in NRRL995	This study
HLW112	Δ*vdoB2* in HLW109	This study
HLW113	Δ*pyrG* in HLW112	This study
HLW114	Δ*dovA* in HLW113	This study
HLW115	Δ*pyrG* in HLW114	This study
HLW116	Δ*vftPS* in HLW115	This study
HLW117	Δ*vftPT* in HLW114	This study
HLW118	Δ*vftAT* in HLW114	This study

*NRRL: ARS Culture Collection

Table S2. Plasmids constructed and used in this study.

Plasmid	Description	Source
pYH-gpdA	*URA*3, *wA* flanking, *gpdA*(*p*), *pyrG*, *ampR*	[4]
pET28a(+)	vector with T7 promoter, 6xHis tag, T7 terminator, kanamycin resistance	Merck, KGaA (Darmstadt, Germany)
p5HY	Two-third of the hph resistance gene at the 5´-end, originated from the pUChph and inserted into pESC-URA. For gene replacement using hph as selection marker	[5]
p3YG	Two-third of the hph resistance gene at the 3´-end, originated from the pUChph and inserted into pESC-URA. For gene replacement using hph as selection marker.	[5]
pHLW28 (pYH-*vdoB2*-Down)	a 315 bp upstream and a 1054 bp downstream PCR fragments of *vdoB2* from genomic DNA of *P. polonicum* NRRL995 inserted in pYH-gpdA	This study
pHLW33 (pYH-*dovA*-Down)	a 331 bp upstream and a 1336 bp downstream PCR fragments of *dovA* from genomic DNA of *P. polonicum* NRRL995 inserted in pYH-gpdA	This study
pHLW35 (pYH-*vftPS*-Down)	a 335 bp upstream and a 1226 bp downstream PCR fragments of *vftPS* from genomic DNA of *P. polonicum* NRRL995 inserted in pYH-gpdA	This study
pHLW75 (pET28a-*vftPT*)	a 1284 bp PCR fragment of *vftPT* from cDNA of *P. polonicum* NRRL995 inserted in pET28a(+)	This study
pHLW77 (pET28a-*vftAT*)	a 1452 bp PCR fragment of *vftAT* from cDNA of *P. polonicum* NRRL995 inserted in pET28a(+)	This study
pHLW114 (p5HY-*vftPT*)	an 898 bp upstream PCR fragment of *vftPT* from genomic DNA of *P. polonicum* NRRL995 inserted in p5HY	This study
pHLW115 (p3YG-*vftPT*)	a 1139 bp downstream PCR fragment of *vftPT* from genomic DNA of *P. polonicum* NRRL995 inserted in p3YG	This study
pHLW116 (p5HY-*vftAT*)	a 929 bp upstream PCR fragment of *vftAT* from genomic DNA of *P. polonicum* NRRL995 inserted in p5HY	This study
pHLW117 (p3YG-*vftAT*)	a 1080 bp downstream PCR fragment of *vftAT* from genomic DNA of *P. polonicum* NRRL995 inserted in p3YG	This study

Table S3. Primers used in this study.

Primer	Oligonucleotide sequence 5'-3'	Function
HLW_pyrG-UP-F-2	caccgcaccattggataatatg	Amplification of the upstream region of *pyrG* from *P. polonicum* NRRL995
HLW_pyrG-UP-R-2	ctcatgtaactacagaggaacgtgcactggctaatcttccgagg	
HLW_pyrG-Dow-F-2	cctcggaagattagccagtgcacgttcctctgtagttacatgag	Amplification of the downstream region of *pyrG* from *P. polonicum* NRRL995
HLW_pyrG-Down-R	cgttatctcgtgagaatatcctcg	
HLW_pyrG_V-F	cgtccaagtcgcaattgac	Screening of Δ*pyrG* transformants
HLW_pyrG_V-R	ctgctggtactgcttagcag	
HLW_penN-U-F	ctaatacgccggacgaacaac	Amplification of the upstream region of *vdoB2* from *P. polonicum* NRRL995
HLW_penN-UP-R	ctctctgcgaccgtccgtctctccgcatgggaccggaatcctgctattg	
HLW_penN-pyrG-gpdA-F	ttgaaacaatagcaggattccggtcccatgcggagagacggacggtc	Amplification of 2/3 from the *pyrG* gene (5'end) for split marker cloning
HLW_pyrG-split-UP-R	cgtagtcaaccgatgccttg	
HLW_penN-300UP-F	catcagtgcctcctctcagacagaatgtgtgttggttacacaggaactc	Amplification of ~300 bp upstream region of *vdoB2* from *P. polonicum* NRRL995
HLW_penN-300UP-R	atactactatgtagtgttacatggcgcagggaccggaatcctgctattg	
HLW_penN-Down-F	ttgaaacaatagcaggattccggtccctgcgccatgtaacactacatag	Amplification of the downstream region of *vdoB2* from *P. polonicum* NRRL995
HLW_penN-Down-R	gtcatagtaaagtgattcgcgtcatgcagcttcgcgataacatcctc	
HLW_pyrG-split-Down-F	cgttaccgtctctgctgatg	Amplification of 2/3 from the *pyrG* gene (3'end) for split marker cloning
HLW_55.1-backbone-F	caacgttcagtgcatcaggac	Screening of Δ*vdoB2* transformants
HLW_55.1-backbone-R	cgacagaattctagagctgaggag	
HLW_penN-V-F	caatgcattgcggccttaag	Screening of Δ*vdoB2* transformants
HLW_penN-V-R	gtccgacgaatgagtgaggtag	Screening of Δ*vdoB2* transformants
HLW_pYH-verA-UP-F	cgcaaccaacttccgtatgtc	Amplification of the upstream region of *dovA* from *P. polonicum* NRRL995
HLW_pYH-verA-UP-R	gccctcctctctgcgaccgtccgtctctccgcatgcttgatgagctcaggaagtgac	
HLW_verA-gpdA-F	cgtaaagtgttgtcgtcacttcctgagctcatcaagcatgcggagagacggacggtc	Amplification of 2/3 from the *pyrG* gene (5'end) for split marker cloning
HLW_verA-300UP-F	catcacgcatcagtgcctcctctcagacagaatcctcgactcgttctaacatacatg	Amplification of ~300 bp upstream region of *dovA* from *P. polonicum* NRRL995
HLW_verA-300UP-R	gtgtaatcaaacacatacaggaatccactagtcgccttgatgagctcaggaagtgac	
HLW_pYH-verA-Down-F	gtaaagtgttgtcgtcacttcctgagctcatcaaggcgactagtggattcctgtatg	Amplification of the downstream region of *dovA* from *P. polonicum* NRRL995
HLW_pYH-verA-Down-R	gtcatagtaaagtgattcgcgtcatggagcagttgttgatggtgcatc	

Table S3. Primers used in this study (continued).

HLW_pHLW01-1_F	gcttgactaacagctaccccgcttgagcagacatcaccatgacaccagaacctattgct	Screening of Δ*dovA* transformants
HLW_pHLW01-1_R	cgcagcatattggctcctac	
verA-knockout-verify-F	gttcaatgatcgatgcagtcctc	Screening of Δ*dovA* transformants
verA-knockout-verify-R	ctagtatgcgccaggttaatg	Screening of Δ*dovA* transformants
HLW_pboA-UP-F	ggatcatacgatcgagtggatc	Amplification of the upstream region of *vftPS* from *P. polonicum* NRRL995
HLW_pboA-UP-R	ctctctgcgaccgtccgtctctccgcatgctattacgcgatgcatgcagc	
HLW_gpdA-pboA-UP-F	ctcacatttgctgcatgcatcgcgtaatagcatgcggagagacggacggtc	Amplification of 2/3 from the *pyrG* gene (5'end) for split marker cloning
HLW_pboA-300UP-F	catcacgcatcagtgcctcctctcagacagaatcagagcagaatcggtgacttag	Amplification of ~300 bp upstream region of *vftPS* from *P. polonicum* NRRL995
HLW_pboA-300UP-R	cttcgctatgcgttgttaagactgccagctattacgcgatgcatgcagc	
HLW_pboA-Down-F	cacatttgctgcatgcatcgcgtaatagctggcagtcttaacaacgcatag	Amplification of the downstream region of *vftPS* from *P. polonicum* NRRL995
HLW_pboA-Down-R	gtcatagtaaagtgattcgcgtcatgcacggcagctattaccatgatc	
HLW_pboA-F	gacgcctggtcaatcttgaac	Screening of Δ*vftPS* transformants
HLW_pboA-R	ctaccgtggtctgcaagatc	
HLW_pboA-V-F	catggtaagcttcctacttcgg	Screening of Δ*vftPS* transformants
HLW_pboA-V-R	cattctgctcgtcgacgaag	Screening of Δ*vftPS* transformants
HLW_pHLW75-F	ggtggacagcaaatgggtcgcggatccatgaccatctcaacaccctcg	Amplification of *vftPT* from *P. polonicum* NRRL995 to construct pHLW75
HLW_pHLW75-R	gcaagcttgtcgacggagctcgctaatggtagtacatggtgagatatggg	
HLW_pHLW77-F	ggtggacagcaaatgggtcgcggatccatgacaggctcaatcctcaag	Amplification of *vftAT* from *P. polonicum* NRRL995 to construct pHLW77
HLW_pHLW76-R	gcaagcttgtcgacggagctcgtcaagaccaaaagggatccgc	
HLW_pHLW114-F	aagaattgttaattaagagctcagatcgatgctgtccaggctatcacag	Amplification of the upstream region of *vftPT* from *P. polonicum* NRRL995 to construct pHLW114
HLW_pHLW114-R	accctcactaaagggcggccgcactagcggaatactggcacaagatcg	
HLW_pHLW115-F	actcactatagggcccgggcgtcgagtgtcggatgagcttgttgatg	Amplification of the downstream region of *vftPT* from *P. polonicum* NRRL995 to construct pHLW115
HLW_pHLW115-R	tagccgcggtaccaagcttactcgactatcaggtgtctacaggcatc	
HLW_pHLW116-F	aagaattgttaattaagagctcagatcggtaacggcgaacagatgag	Amplification of the upstream region of *vftAT* from *P. polonicum* NRRL995 to construct pHLW116
HLW_pHLW116-R	accctcactaaagggcggccgcactaggatgctgtccaggctatcacag	
HLW_pHLW117-F	actcactatagggcccgggcgtcgagctgactacacctcagtaggtc	Amplification of the downstream region of *vftAT* from *P. polonicum* NRRL995 to construct pHLW117
HLW_pHLW117-R	tagccgcggtaccaagcttactcgactagctctgcatggtgatcac	

Table S3. Primers used in this study (continued).

5-HYGsplit-R	tcgaagtagcgcgtctgc	Amplification of 2/3 from the *hph* gene (5'end) for split marker cloning
3-HYGsplit-F	ttggggaattcagcgagagc	Amplification of 2/3 from the *hph* gene (3'end) for split marker cloning
HLW_VftPT-F	cgactatcgaagttgccgag	Screening of Δ*vftPT* transformants
HLW_VftPT-R	ctgatggtccgtagcgacatc	
HLW_VftPT-V-F	cattgcaattggcatgaagtcg	Screening of Δ*vftPT* transformants
HLW_VftPT-V-R	gtcgagtgtggacggatacag	Screening of Δ*vftPT* transformants
HLW_VftAT-F	cgacttcatgccaattgcaatg	Screening of Δ*vftAT* transformants
HLW_VftAT-R	cgcatgcatgtatctagtccac	
HLW_VftAT-V-F	ctcatattgccgatgaacgctg	Screening of Δ*vftAT* transformants
HLW_VftAT-V-R	cgagaatggcatcaacaaggc	Screening of Δ*vftAT* transformants

Table S4. Proteins encoded by the *vft* and *vdo2* cluster in *Penicillium polonicum* NRRL995.

P. polonicum NRRL995	Length (aa)	Location at JAPDKX010000015.1	Identity	Putative function
VftPS	2047	(20001-20003, 20001-22220, 22282-22371, 22426-22700, 22768-25728, 25793-26387)	53.2% to PboA (A0A0C1E6T8.1)	Nonribosomal peptide synthetase
VftPT	428	(29977-29979, 29977-30076, 30160-31343)	62.2% to RoqD (B6HJU1.1)	Prenyltransferase
VftAT	484	(32068-33519, 33517-33519)	46.1% to PboB (A0A0C1E2S7.1)	Acetyltransferase

P. polonicum NRRL995	Length (aa)	Location at JAPDKX010000055.1	Identification	Putative function
VdoA2	748	(26247-26492, 26547-28544)	91.1% to VdoA (UHY14092.1)	Hemocyanin-like
VdoB2	2680	(17262-25301, 25299-25301)	90.3% to VdoB (UHY14093.1)	Nonribosomal peptide synthetase
VdoC2	297	(14475-15365, 15363-15365)	94.9% to VdoC (UHY14095.1)	Fe^{II}/2OG dependent oxygenase
VdoD2	496	(10774-10776, 10774-11115, 11196-12341)	95.5% to VdoD (UHY14094.1)	Cytochrome P450

Table S5. Catalytic efficiency (k_{cat}/K_M ($s^{-1}\cdot M^{-1}$)) of other reported PTs.

	FtmPT1[6]	BrePT[7]	CdpC2PT[8]	CdpC7PT[9]
DMAPP	-	4992	8765	847.5
**6**	-	134	98	65.3
**6a**	6407	27	-	70.0
**6b**	-	40	35	126.6
**6c**	-	-	1815	139.3
**6d**	25312	8639	-	-
**6e**	2722	37	36	12.3
**6f**	4756	-	-	-
**6g**	4078	25	-	-

See Table 1 in the main text for the data of VftPT. The data are adopted from previous publications as indicated.

Table S6. The ^{1}H NMR (500 MHz) data of verrucofortine (**1**) in $CDCl_3$.

Compd.

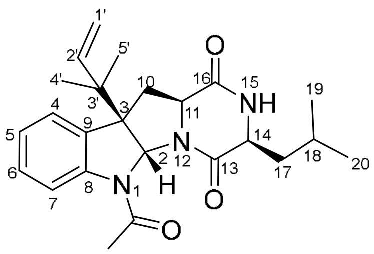

verrucofortine (**1**)

Position	δ_H, multi., J	Position	δ_H, multi., J
2	5.95, s	17	1.54, m
4	7.28, d, 7.6	18	1.68, m
5	7.14, br t, 7.6	19	0.99, d, 6.6
6	7.31, br t, 7.6	20	0.90, d, 6.6
7	7.99, br s	1’	5.13, d, 10.9
10	2.60, br t, 5.9		5.10, d, 9.3
	2.39, dd, 12.6, 11.2	2’	5.79, dd, 17.2, 10.9
11	3.85, dd, 11.2, 5.6	4’	1.15, s
14	3.96, dd, 10.1, 3.1	5’	0.97, s
15	5.98, s	N_1-$COCH_3$	2.63, s

The NMR data of **1** correspond to those of verrucofortine (fructigenine B), as reported previously.[10,11]

Table S7. The ^{1}H NMR (500 MHz) data of viridicatol (**2**), viridicatin (**3**) and 3-*O*-methylviridicatin (**4**) in DMSO-d_6.

Compd.	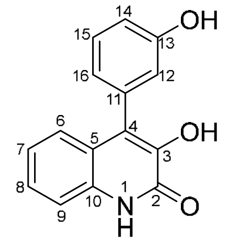	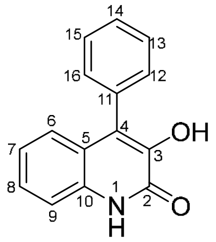	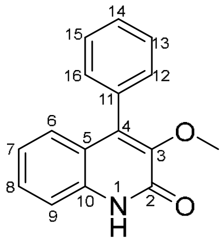
	viridicatol	viridicatin	3-*O*-methylviridicatin
Position	δ_H, multi., *J*	δ_H, multi., *J*	δ_H, multi., *J*
1 (NH)	12.17, br s	12.20, br s	12.08, br s
3	9.09, br s (OH)	9.14, br s (OH)	3.67, s (OCH_3)
6	7.33[a]	7.06[c], br d, 8.1	6.97, br d, 8.0
7	7.07, br t, 8.4	7.07[c], br t, 8.1	7.07, br t, 8.0
8	7.10, br t, 8.4	7.34[d], br t, 8.1	7.41, br t, 8.0
9	7.31[a], br d, 8.4	7.34[d]	7.37, br d, 8.0
12	6.72[b], s	7.34[d]	7.31, br d, 7.6
13	9.49, br s (OH)	7.51, br t, 7.8	7.51, br t, 7.6
14	6.82, br d, 8.0	7.44, br t, 7.8	7.47, br t, 7.6
15	7.29[a], br t, 8.0	7.51, br t, 7.8	7.51, br t, 7.6
16	6.73[b], br d, 8.0	7.34[d]	7.31, br d, 7.6

[a, b, c, d]: Signals labeled with the same letters overlap with each other.

The NMR data of **2**, **3**, and **4** correspond to those reported for viridicatol, viridicatin, and 3-*O*-methylviridicatin, respectively.[12,13]

Table S8. The ^{1}H NMR (400 MHz) data of *cyclo*-L-Trp-L-Leu (**6**) and *allo*-brevicompanine B (**7**) in $CDCl_3$.

Compd.	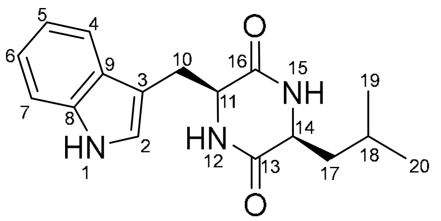	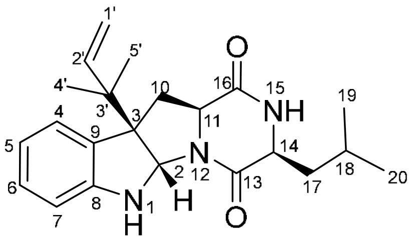
	cyclo-L-Trp-L-Leu (**6**)	*allo*-brevicompanine B (**7**)
Position	δ_H, multi., *J*	δ_H, multi., *J*
1	8.16, s	-
2	7.11, br s	5.50, s
4	7.64, d, 7.8	7.15 d,7.7
5	7.15, t, 7.6	6.76, br t, 7.6
6	7.23, t, 7.6	7.10, br t, 7.6
7	7.39, d, 7.8	6.58, d, 7.7
10	3.48, dd, 14.7, 3.3	2.55, dd, 12.8, 6.6
	3.22, dd, 14.7, 8.4	2.46, dd, 12.8, 10.8
11	4.32, m	3.94, m
12	6.02, s	-
14	3.90, m	3.97, m
15	5.98, s	5.76, s
17	1.51, m	2.01, m
	1.49, m	1.68, m
18	0.95, m	1.57, m
19	0.83, d, 6.3	0.99, d, 6.6
20	0.81, d, 6.3	0.91, d, 6.6
1’	-	5.12, dd, 10.8, 1.1
	-	5.08, dd, 17.4, 1.1
2’	-	5.97, dd, 17.4, 10.8
4’	-	1.01, s
5’	-	1.12, s

The NMR data of **6** and **7** correspond to those reported for *cyclo*-L-Trp-L-Leu and *allo*-brevicompanine B (*C3β*-prenylated *cyclo*-L-Trp-L-Leu), as reported previously.[14–16]

Table S9. The ^{1}H NMR (400 MHz) data of **7a** and **7b** in $CDCl_3$.

Compd.	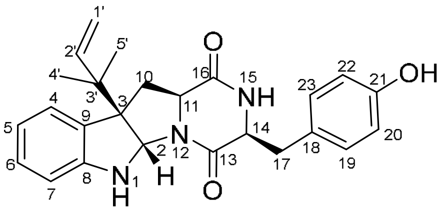 **7a**	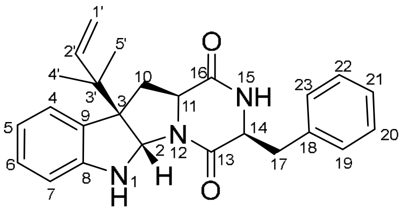 **7b**
Position	δ_H, multi., *J*	δ_H, multi., *J*
2	5.52, s	5.54 [a], s
4	7.13, d, 7.7	7.14, d, 7.6
5	6.74, t, 7.4	6.76, td, 7.6, 1.2
6	7.10, t, 7.4	7.11, td, 7.6, 1.2
7	6.58, d, 7.7	6.59, d, 7.6
10	2.51, dd, 12.5, 6.3	2.52, dd, 12.6, 6.2
	2.36, t, 11.2	2.40, t, 11.2
11	3.93, dd, 9.9, 6.3	3.93, ddd, 11.3, 6.2, 1.8
14	4.13, d, 8.2	4.21, ddd, 10.8, 3.5, 1.7
15	5.79, s	5.54 [a], s
17	3.44, dd, 14.5, 4.0	3.60, dd, 14.4, 3.4
	2.77, dd, 14.5, 10.1	2.79, dd, 14.4, 10.7
19	7.03, d, 8,4	7.20, d, 7.4
20	6.76, d, 8,4	7.34, t, 7.2
21	-	7.29, t, 7.1
22	6.76, d, 8,4	7.34, t, 7.2
23	7.03, d, 8,4	7.20, d, 7.4
1’	5.12, d, 10.8	5.13, dd, 10.8, 1.1
	5.06, d, 17.5	5.08, dd, 17.4, 1.1
2’	5.93, dd, 17.3, 10.6	5.96, dd, 17.4, 10.8
4’	0.98, s	1.01, s
5’	1.09, s	1.11, s

[a]: Overlapping signals.

The NMR data of **7a** and **7b** correspond to those reported for *C3β*-prenylated *cyclo*-L-Trp-L-Tyr and *C3β*-prenylated *cyclo*-L-Trp-L-Phe, respectively.[16]

Table S10. The ^{1}H NMR (400 MHz) data of **7c** and **7d** in $CDCl_3$.

Compd.	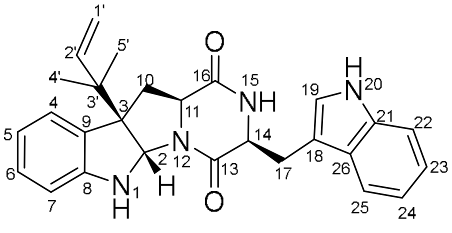 **7c**	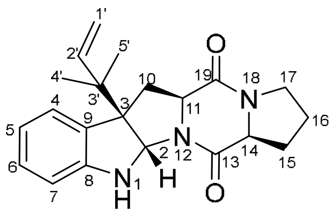 **7d**
Position	δ_H, multi., *J*	δ_H, multi., *J*
2	5.55, s	5.46, s
4	7.14 [a]	7.16, d, 7.6
5	6.75, td, 7.6, 1.1	6.76, td, 7.6, 1.1
6	7.08 [b]	7.10, td, 7.6, 1.1
7	6.61, d, 7.6	6.58, d, 7.6
10	2.51, dd, 12.6 ,6.3	2.49, dd, 12.9, 10.5
	2.41, t, 11.0	2.54, dd, 12.9, 6.8
11	3.91, ddd, 11.0, 6.4, 1.9	3.98, dd, 10.5, 6.8
14	4.30, ddd, 11.0, 3.8, 1.9	4.05, t, 9.6
15	5.70, s	2.31, m
	–	2.12, m
16	–	2.04, m
	–	1.89, m
17	3.74, dd, 15.1, 2.5	3.55, m
	2.97, dd, 15.1, 11.0	3.50, m
19	7.10 [b]	–
20	8.19, s	–
22	7.38, d, 8.2	–
23	7.22, td, 7.1, 1.2	–
24	7.12 [a]	–
25	7.55, d, 8.2	–
1’	5.12, dd, 10.8, 1.1	5.11, dd, 10.8, 1.1
	5.07, dd, 17.4, 1.1	5.07, dd, 17.4, 1.1
2’	5.96, dd, 17.4, 10.8	5.99, dd, 17.4, 10.8
4’	1.00, s	1.11, s
5’	1.11, s	1.00, s

[a,b]: Signals labeled with the same letters overlap with each other.

The NMR data of **7c** and **7d** correspond to those reported for *C3β*-prenylated *cyclo*-L-Trp-L-Trp and *C3β*-prenylated *cyclo*-L-Trp-L-Pro, respectively.[16,17]

Table S11. The ^{1}H NMR (400 MHz) data of **7e** in $CDCl_3$ and **7f** in CD_3OD.

Compd.	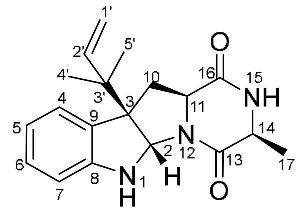	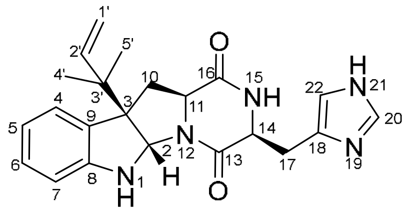
	**7e**	**7f**
Position	δ_H, multi., *J*	δ_H, multi., *J*
2	5.50, s	5.52, s
4	7.15, d, 7.4	7.17, d, 7.8
5	6.76, td, 7.4, 1.0	6.70, t, 7.6
6	7.10, td, 7.4, 1.0	7.05, t, 7.6
7	6.58, d, 7.4	6.57, d, 7.8
10	2.46, dd, 12.7, 10.9	2.42, dd, 12.2, 5.7
	2.54, dd, 12.7, 6.5	2.15, dd, 12.2, 11.8
11	3.95, dd, 11.1, 6.7	3.93, dd, 11.8, 6.3
14	4.03, qd, 6.9, 1.8	4.35, br s
15	6.25, br.s	–
17	1.46, d, 6.9	3.14 [a]
	–	3.17 [a]
20	–	7.76, s
22	–	6.92, s
1’	5.12, dd, 10.9, 1.2	5.11, d, 10.8
	5.08, dd, 17.4, 1.2	5.06, d, 17.4
2’	5.98, dd, 17.4, 10.9	5.95, dd, 17.4, 10.8
4’	1.12, s	0.94, s
5’	1.01, s	1.07, s

[a]: Overlapping signals.

The NMR data of **7e** and **7f** correspond to those reported for *C3β*-prenylated *cyclo*-L-Trp-L-Ala and *C3β*-prenylated *cyclo*-L-Trp-L-His, respectively.[18–20]

Table S12. The ^{1}H NMR (400 MHz) data of **7g** in $CDCl_3$.

Compd.	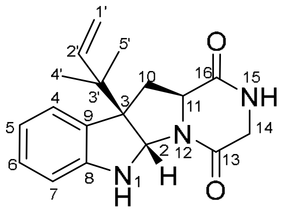 **7g**
Position	δ_H, multi., *J*
2	5.54, s
4	7.15, d, 7.6
5	6.76, td, 7.6, 1.2
6	7.10, td, 7.6, 1.2
7	6.59, d, 7.6
10	2.55, dd, 12.6, 6.2
	2.44, t, 12.6
11	3.94, ddd, 11.4, 6.3, 2.3
14	4.02, dd, 17.1, 2.1
	3.88, dd, 17.1, 4.0
15	6.65, br.s
1’	5.13, dd, 10.8, 1.2
	5.08, dd, 17.2, 1.2
2’	5.97, dd, 17.2, 10.8
4’	1.00, s
5’	1.12, s

The NMR data of **7g** correspond to those for *C3β*-prenylated *cyclo*-L-Trp-Gly, as reported previously .[12]

Supplementary Figures

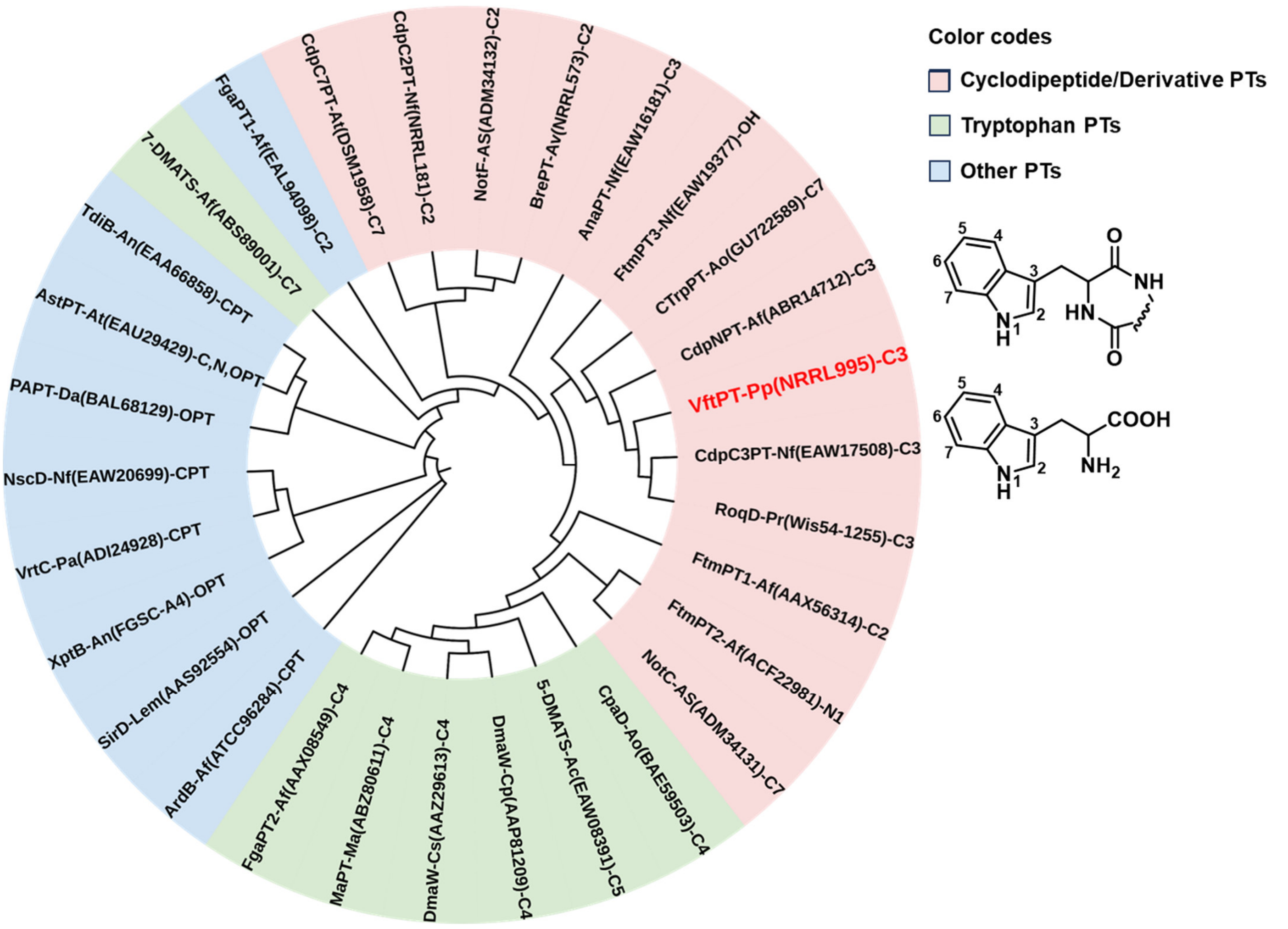

Figure S1. Phylogenetic tree of prenyltransferases.

The sequence alignments and phylogenetic analysis were performed with MEGA (version 12) using the maximum likelihood method. The prenylation positions of cyclodipeptides, their derivatives, and tryptophan are indicated as labeled in the structures. For other PTs, only the prenylation type (C-, O-, or N-prenylation) is shown. VftPT is located in the branch of several *C3*-prenyltransferases.

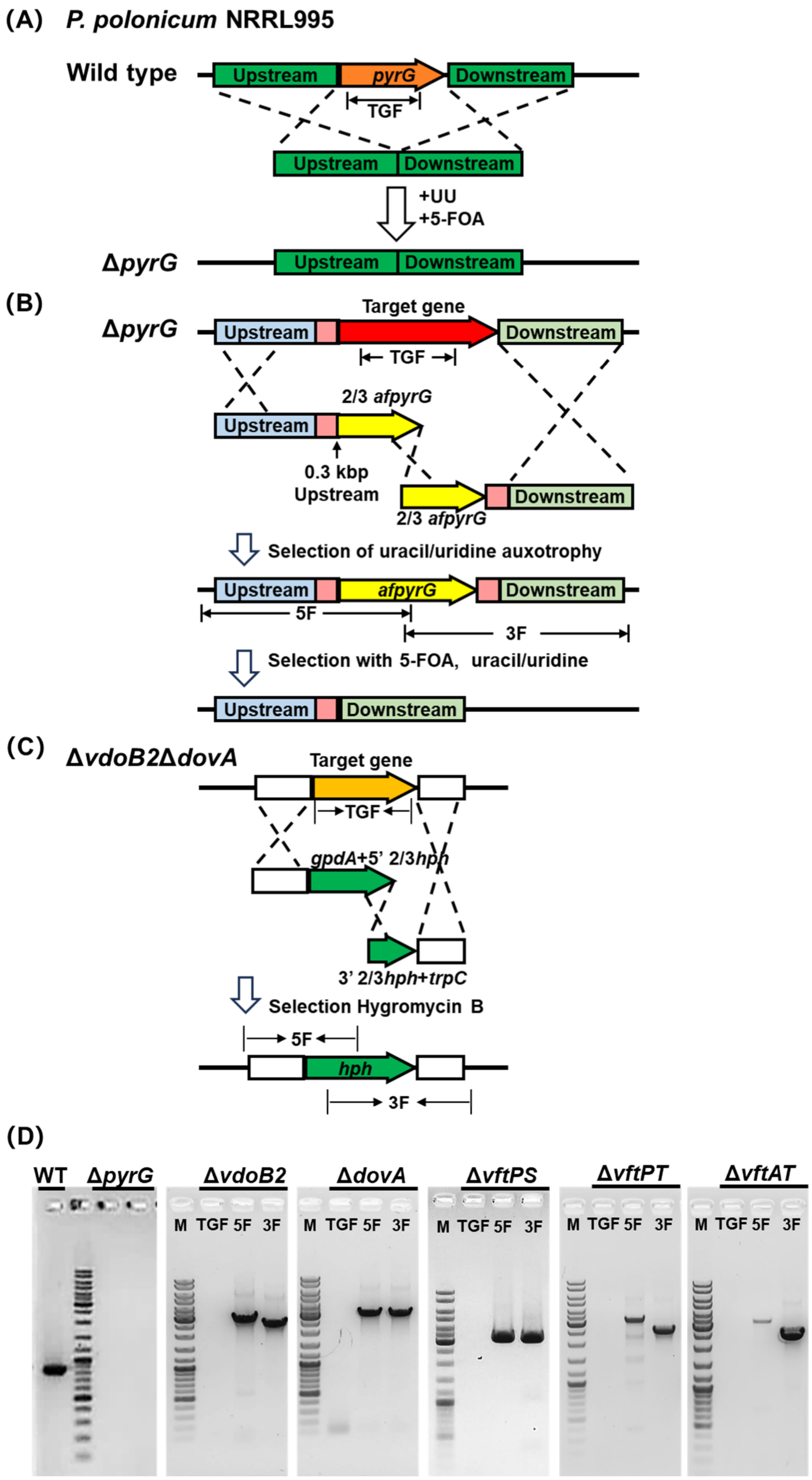

Figure S2. Target gene deletion and PCR verification for *P. polonicum* strain.
(A) Schematic diagram of *pyrG* gene deletion. (B) Split-marker strategy for target gene disruption based on auxotrophy and marker recycling. (C) Split-marker strategy for target gene disruption with hygromycin B. (D) PCR verification of target gene deletion.

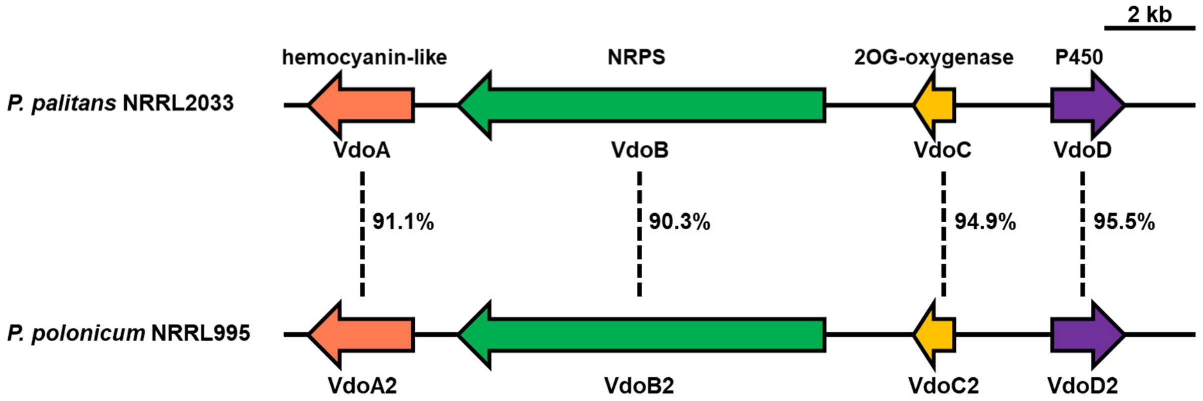

Figure S3. Comparison of viridicatol biosynthetic gene clusters in *P*. *palitans* and *P*. *polonicum*. The sequence identities on the amino acid level are given in percent.

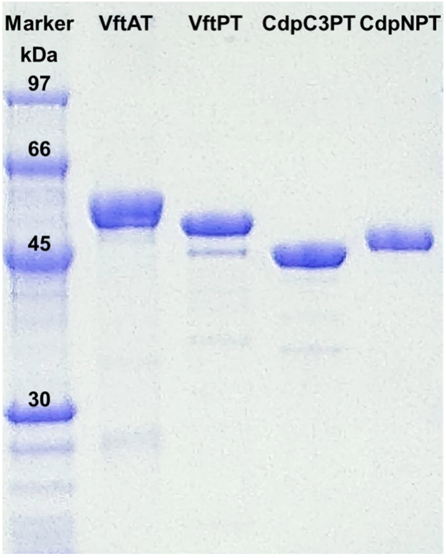

Figure S4. SDS-PAGE of the purified VftAT, VftPT, CdpC3PT, and CdpNPT.

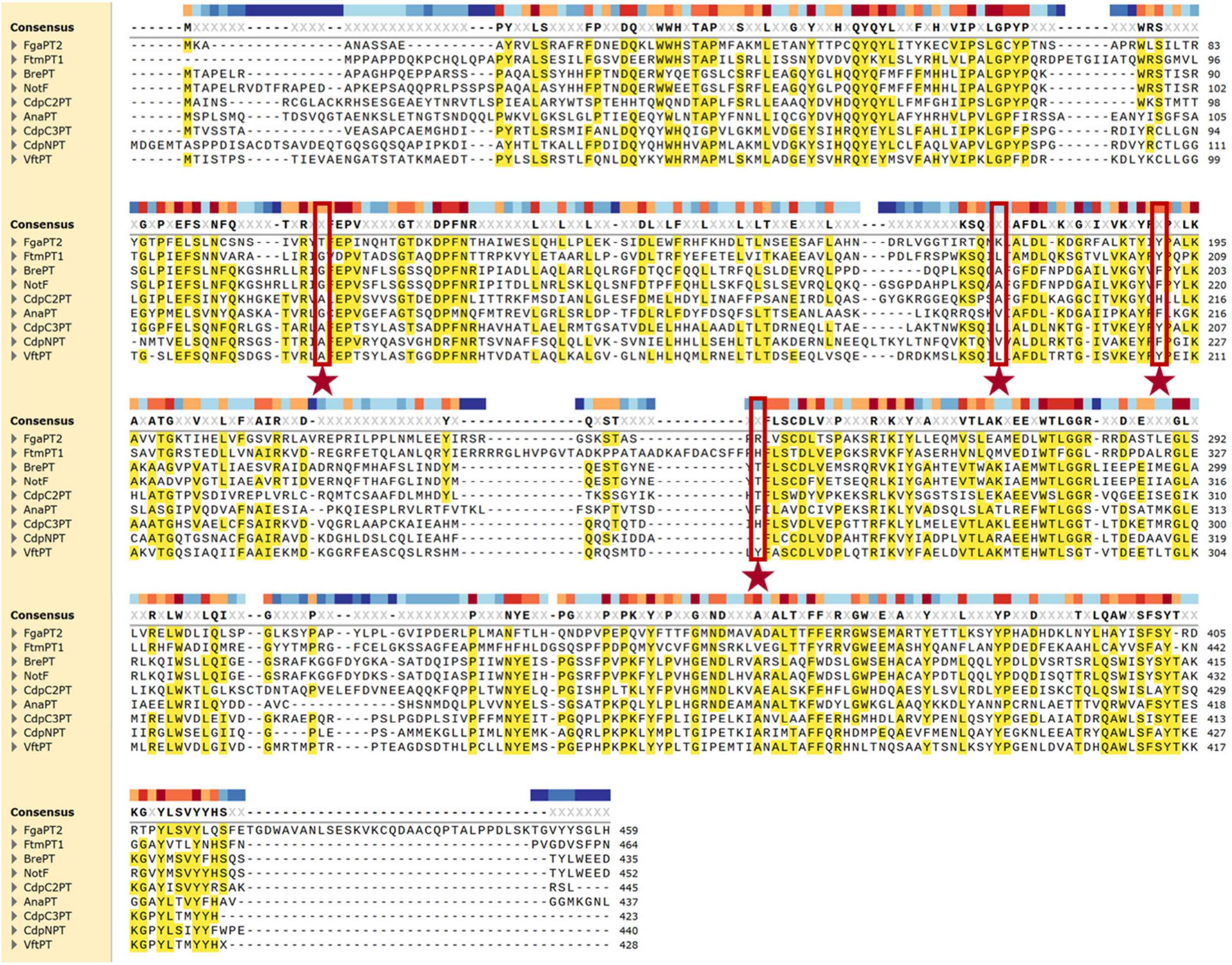

Figure S5. Sequence alignments of FgaPT2, FtmPT1, NotF, BrePT, CdpC2PT, AnaPT, CdpC3PT, CdpNPT, and VftPT.

The key positions T102, K174, and R244 in FgaPT2 (corresponding to G115, L187, and H279 in FtmPT1, A119, L190, and Y256 VftPT) and Y191 in FgaPT2 (corresponding to Y205 in FtmPT1 and Y207 in VftPT) are marked with ★.

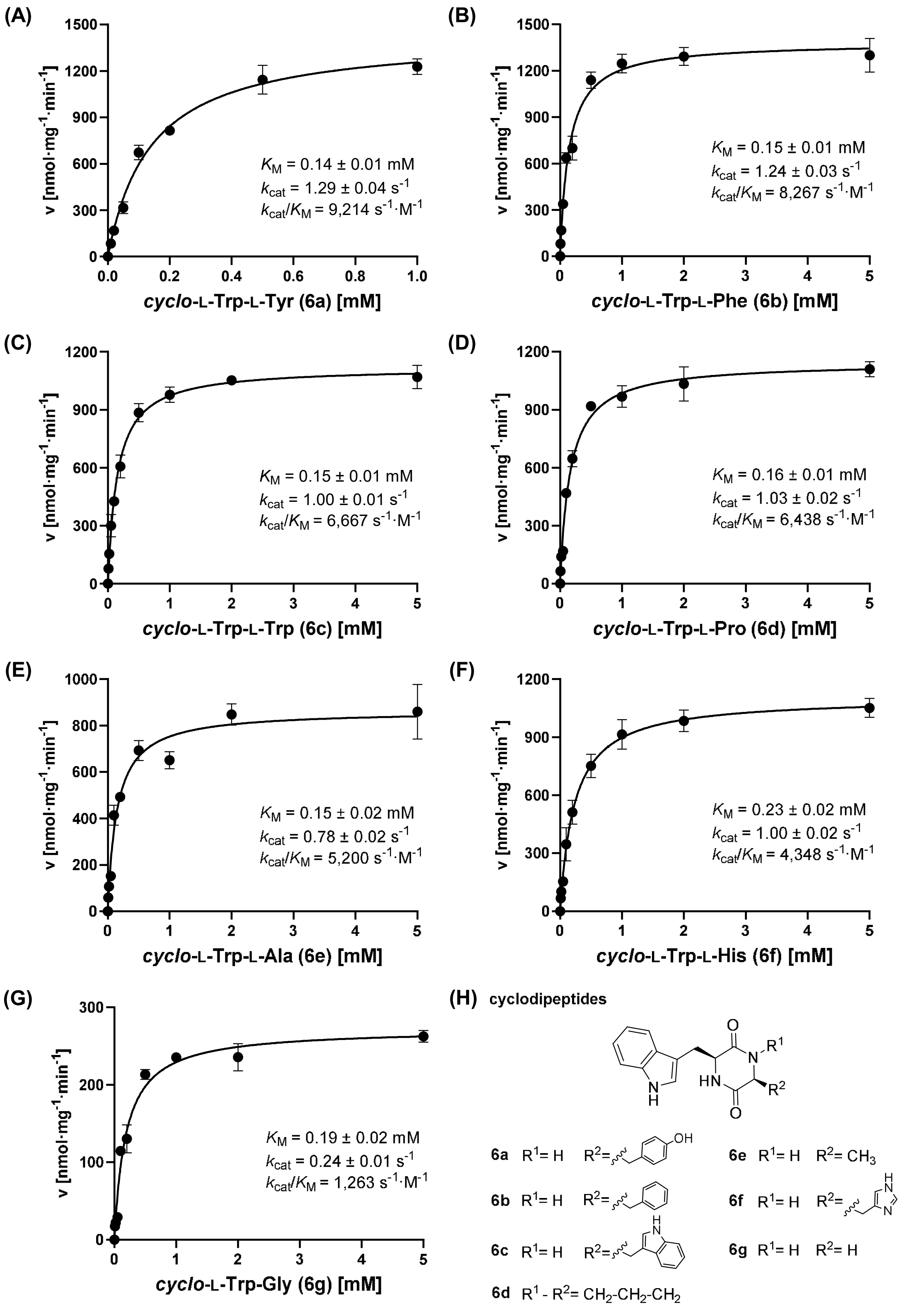

Figure S6. Kinetic parameters of VftPT toward selected cyclodipeptides (**6a**–**6g**).

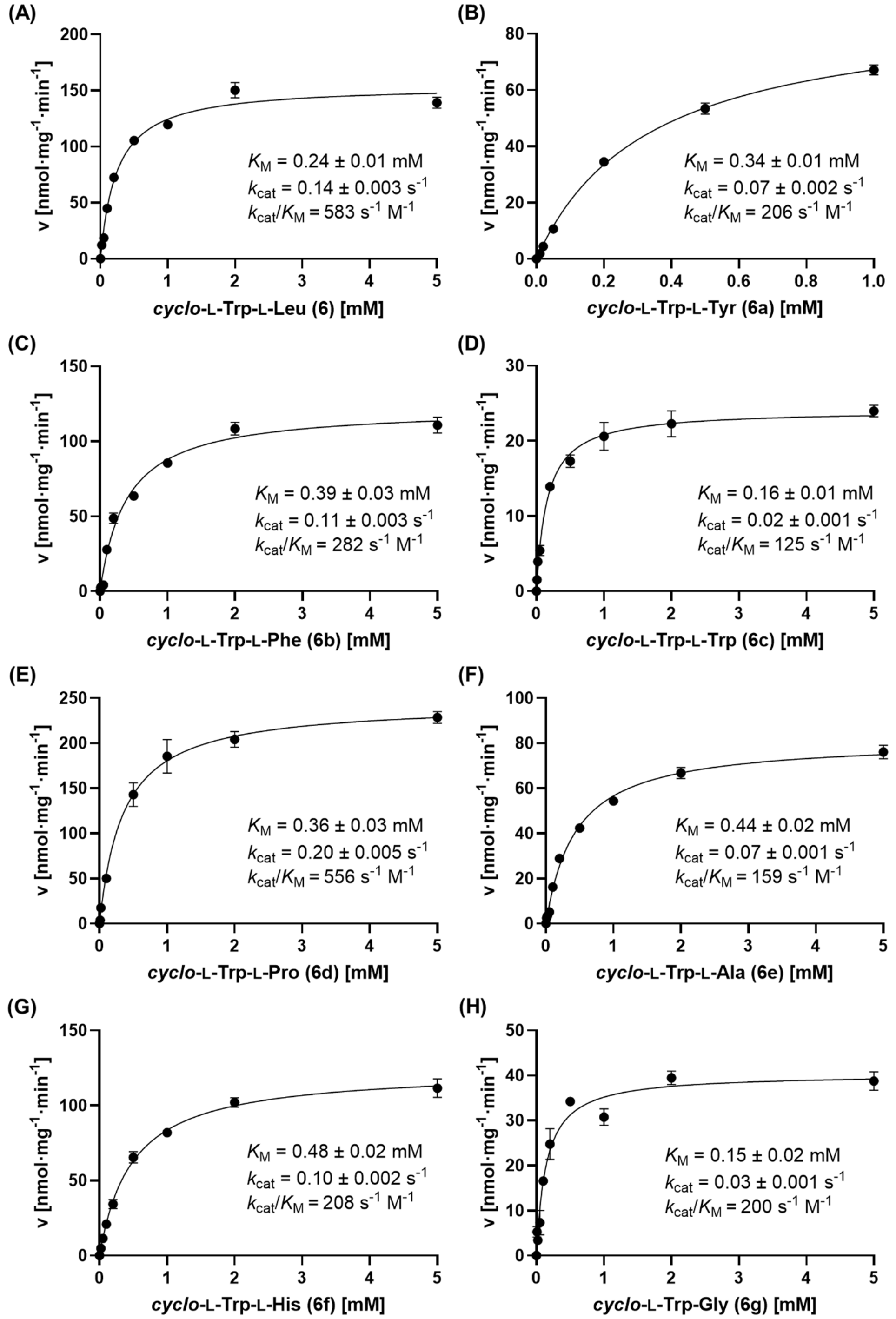

Figure S7. Kinetic parameters of CdpC3PT toward selected cyclodipeptides (**6** and **6a**–**6g**). See Figure S6 for structures.

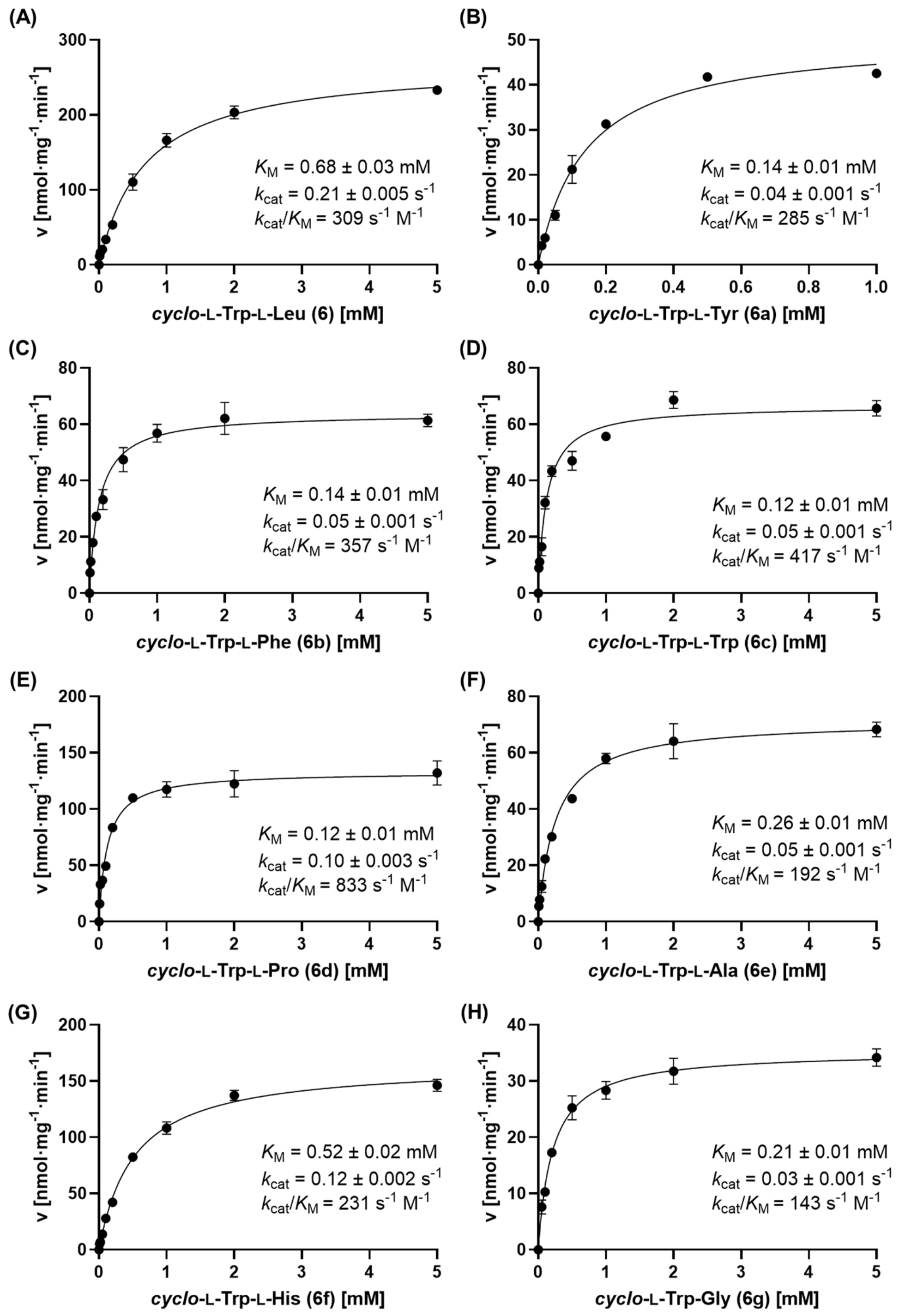

Figure S8. Kinetic parameters of CdpNPT toward selected cyclodipeptides (**6** and **6a**–**6g**). See Figure S6 for structures.

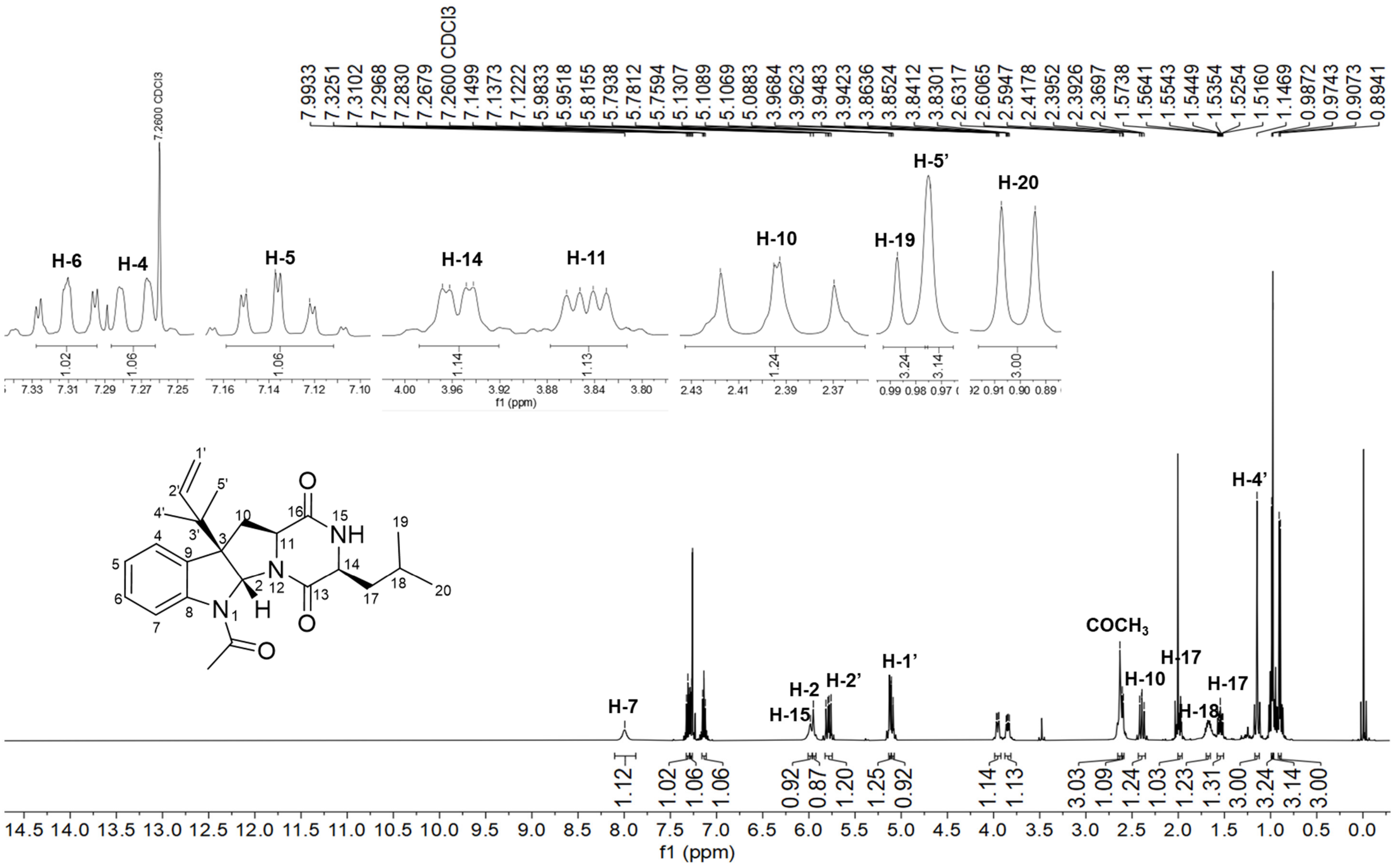

Figure S9. ^{1}H NMR spectrum of verrucofortine (**1**) in $CDCl_3$ (500 MHz).

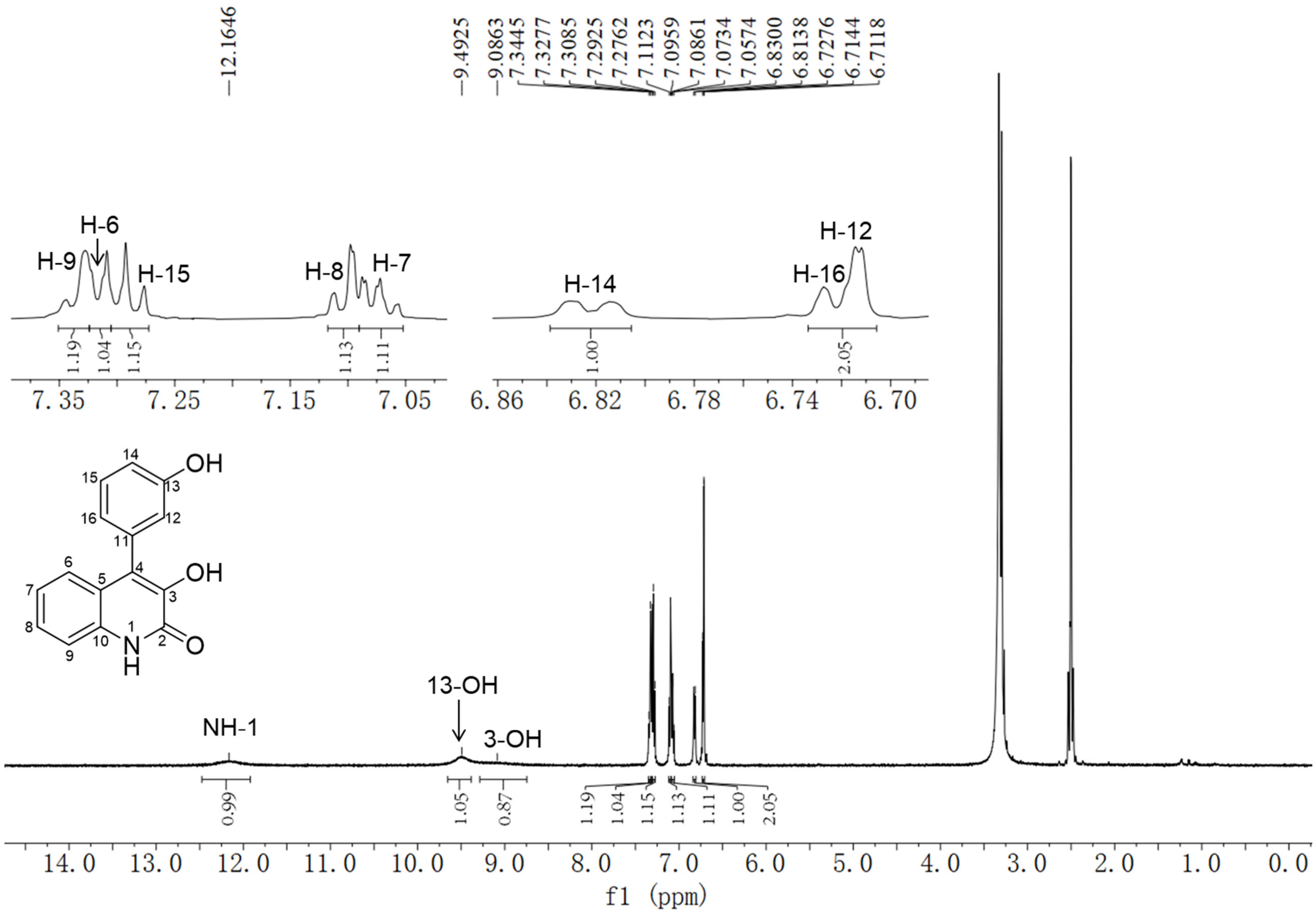

Figure S10. ^{1}H NMR spectrum of viridicatol (**2**) in DMSO-d_6 (500 MHz).

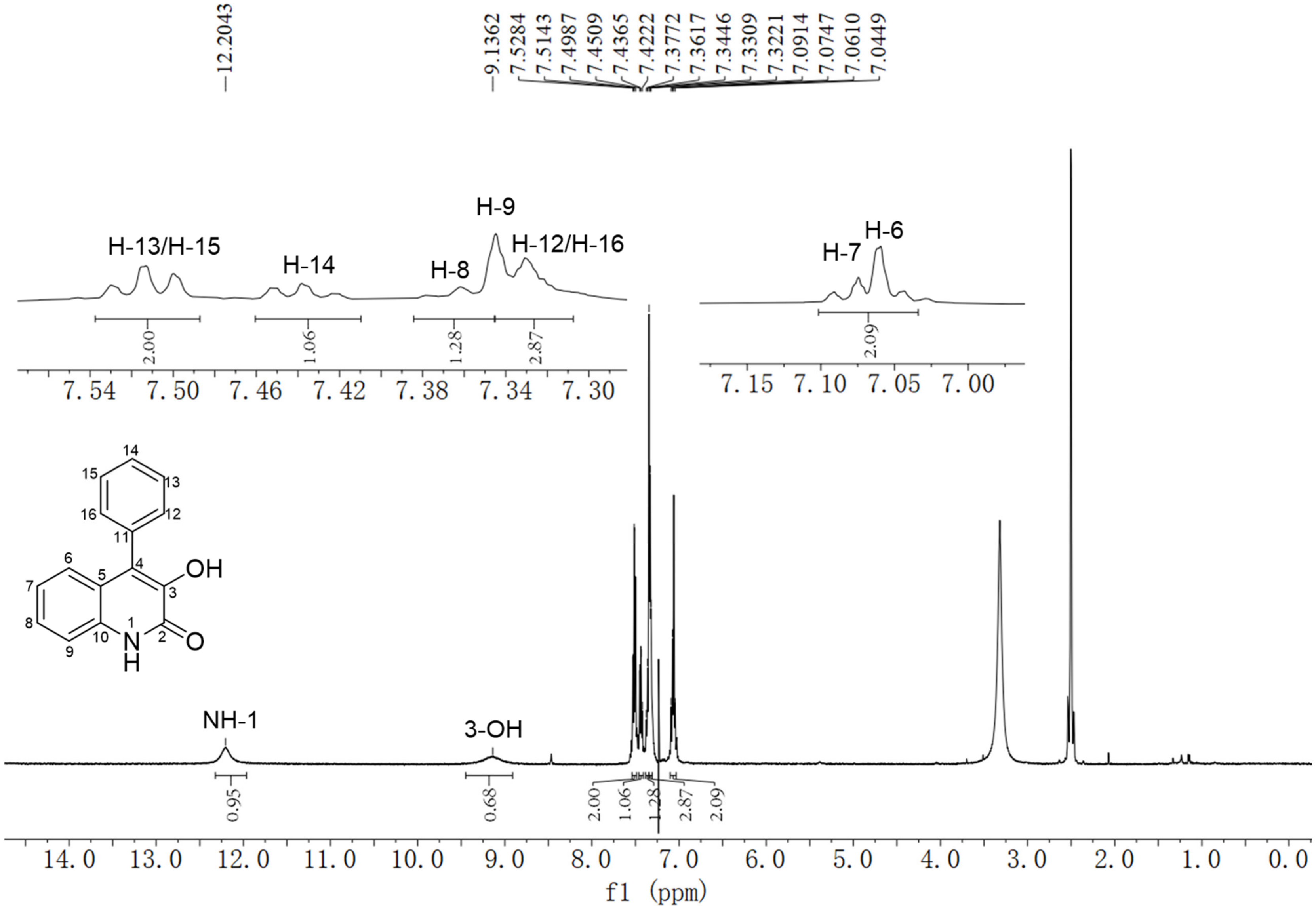

Figure S11. ^{1}H NMR spectrum of viridicatin (**3**) in DMSO-d_6 (500 MHz).

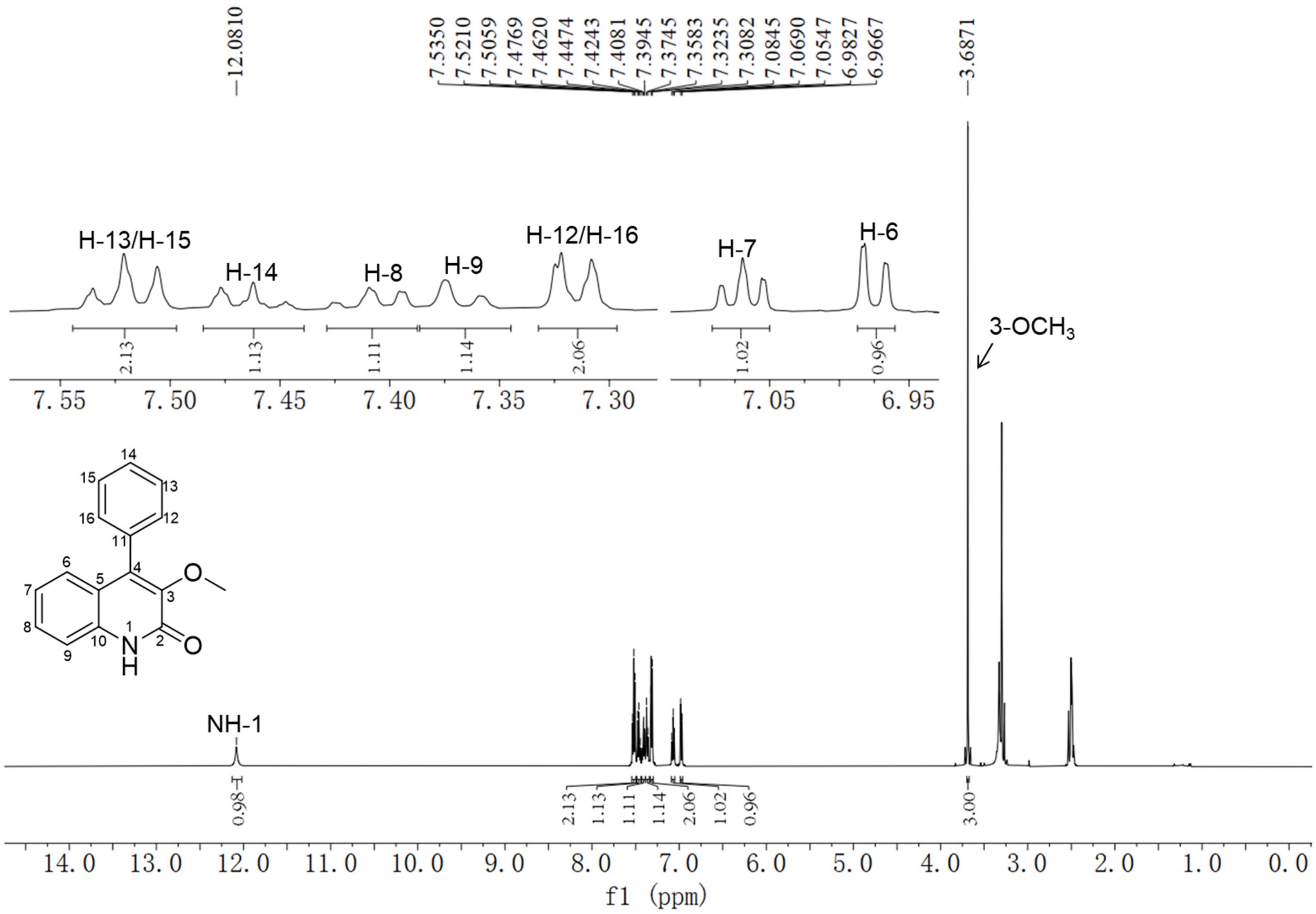

Figure S12. ^{1}H NMR spectrum of 3-*O*-methylviridicatin (**4**) in DMSO-d_6 (500 MHz).

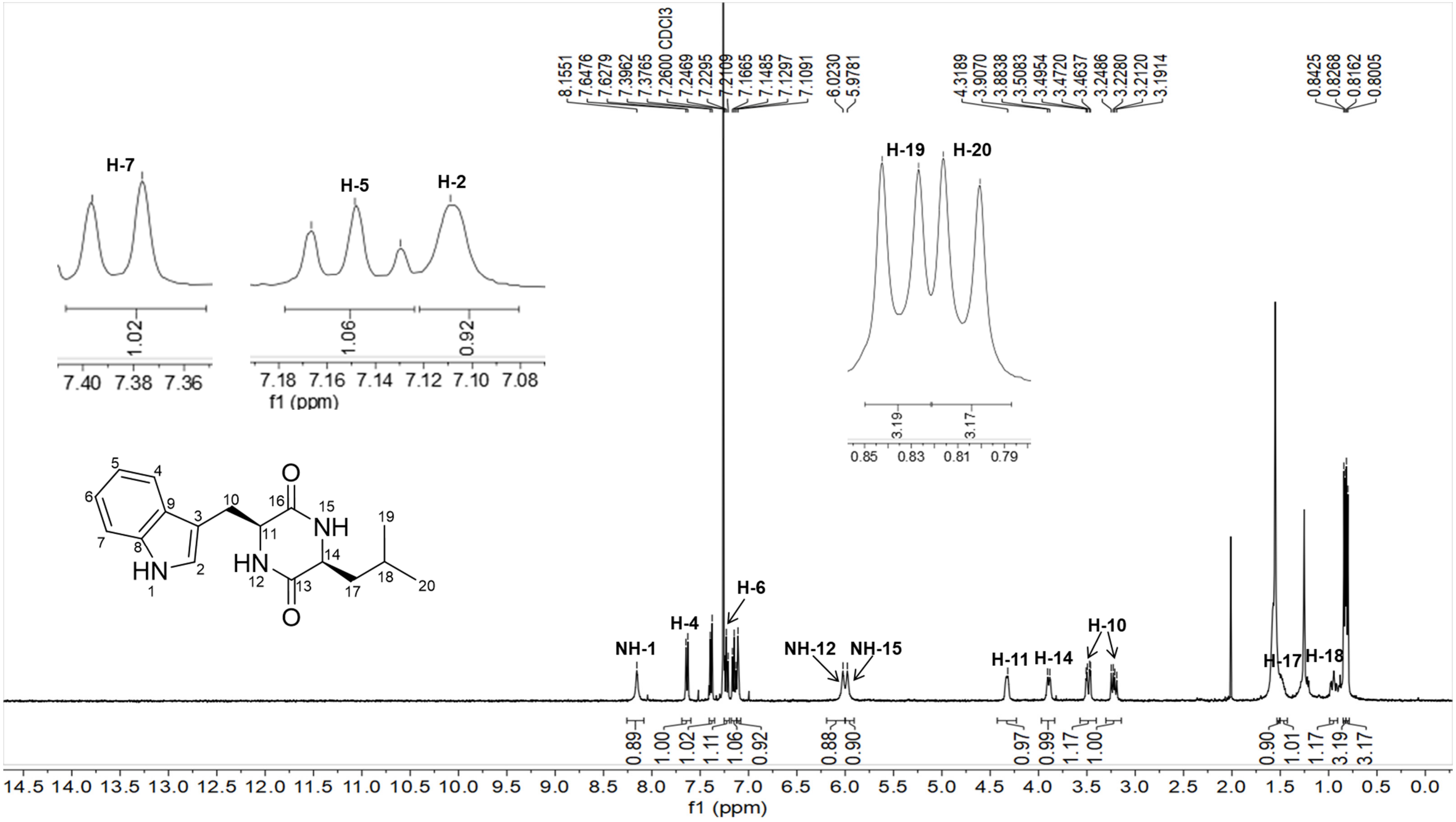

Figure S13. ^{1}H NMR spectrum of *cyclo*-L-Trp-L-Leu (**6**) in $CDCl_3$ (400 MHz).

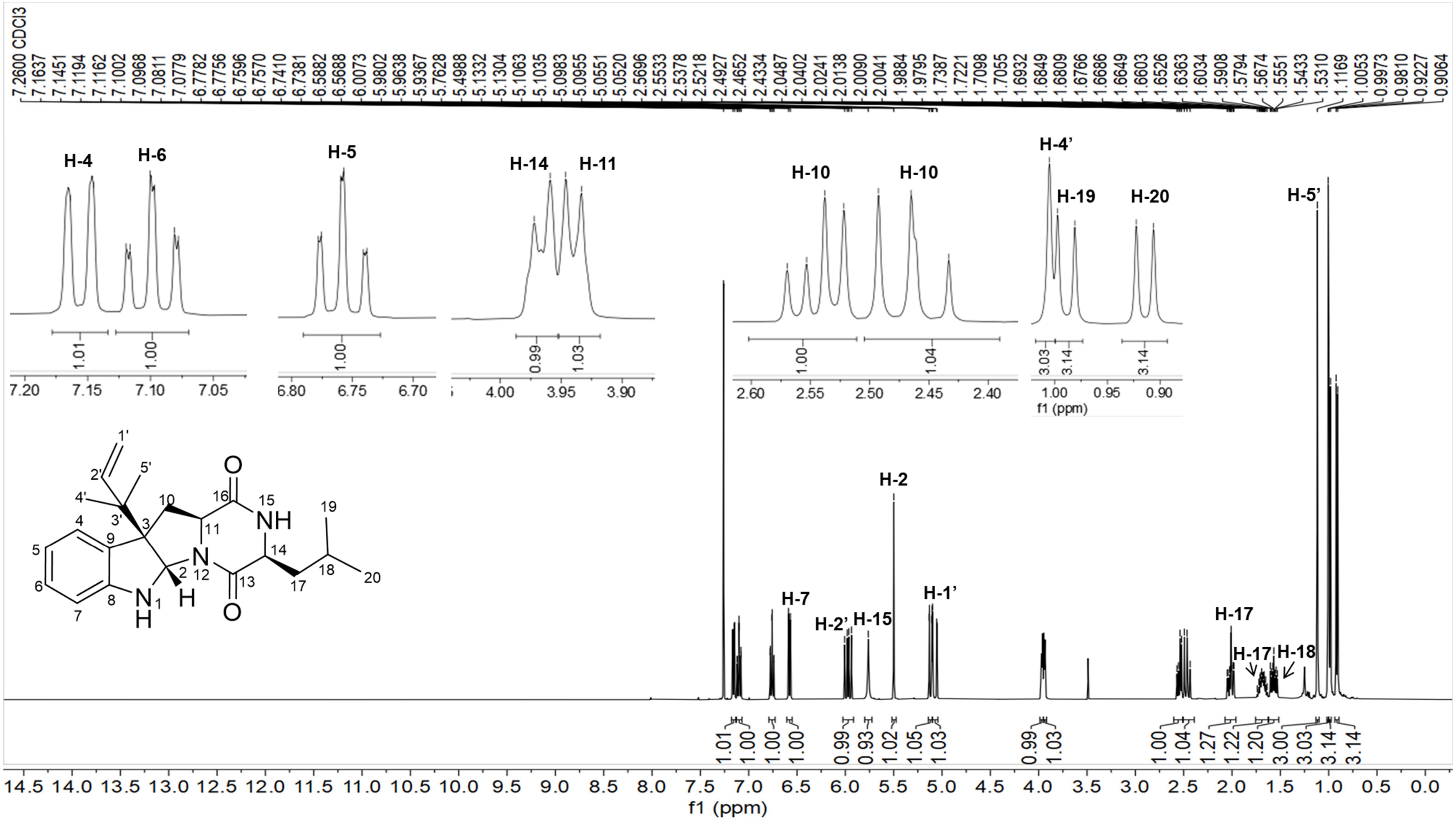

Figure S14. ^{1}H NMR spectrum of *allo*-brevicompanine B (**7**) in $CDCl_3$ (400 MHz).

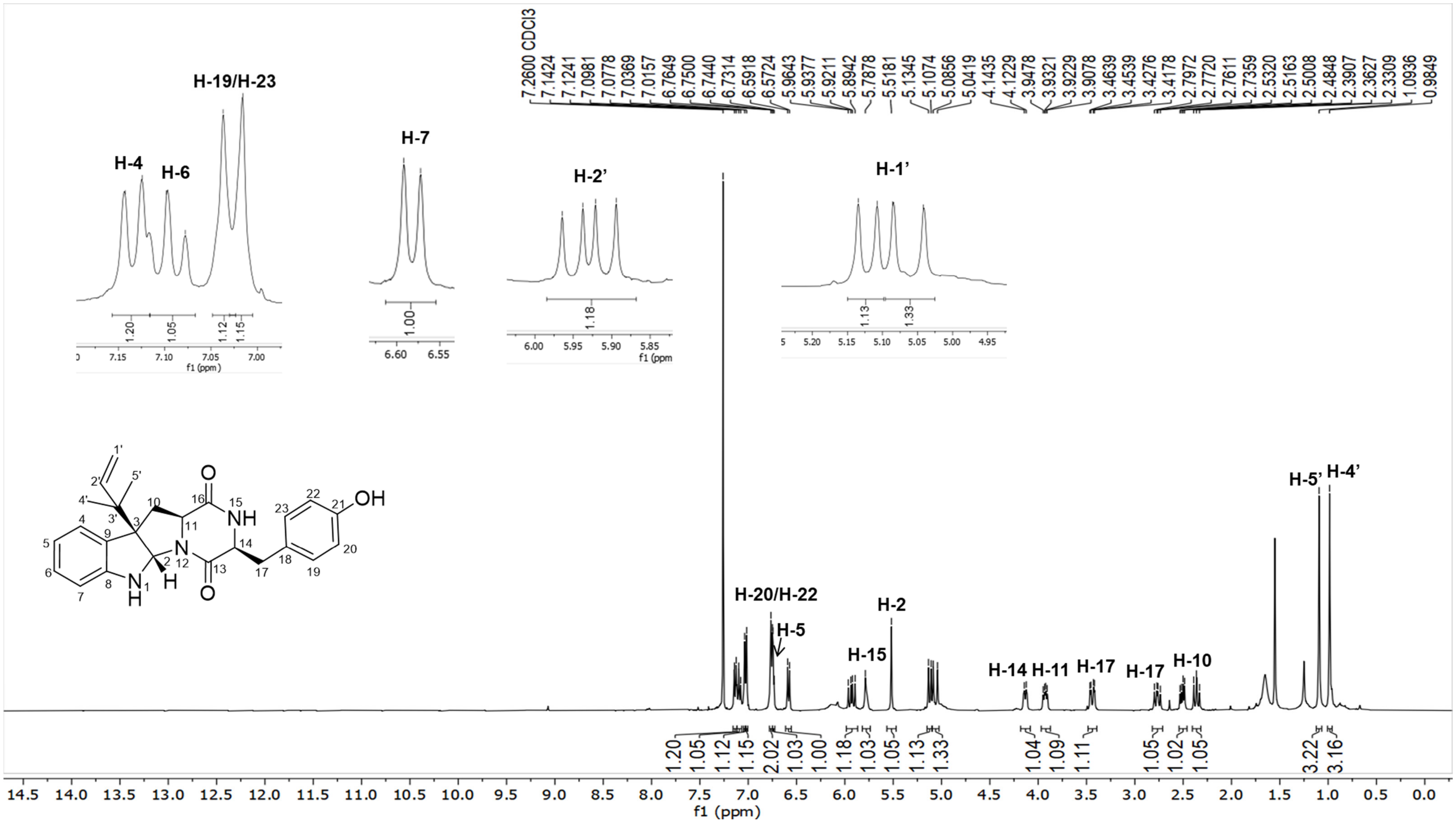

Figure S15. ^{1}H NMR spectrum of **7a** in $CDCl_3$ (400 MHz).

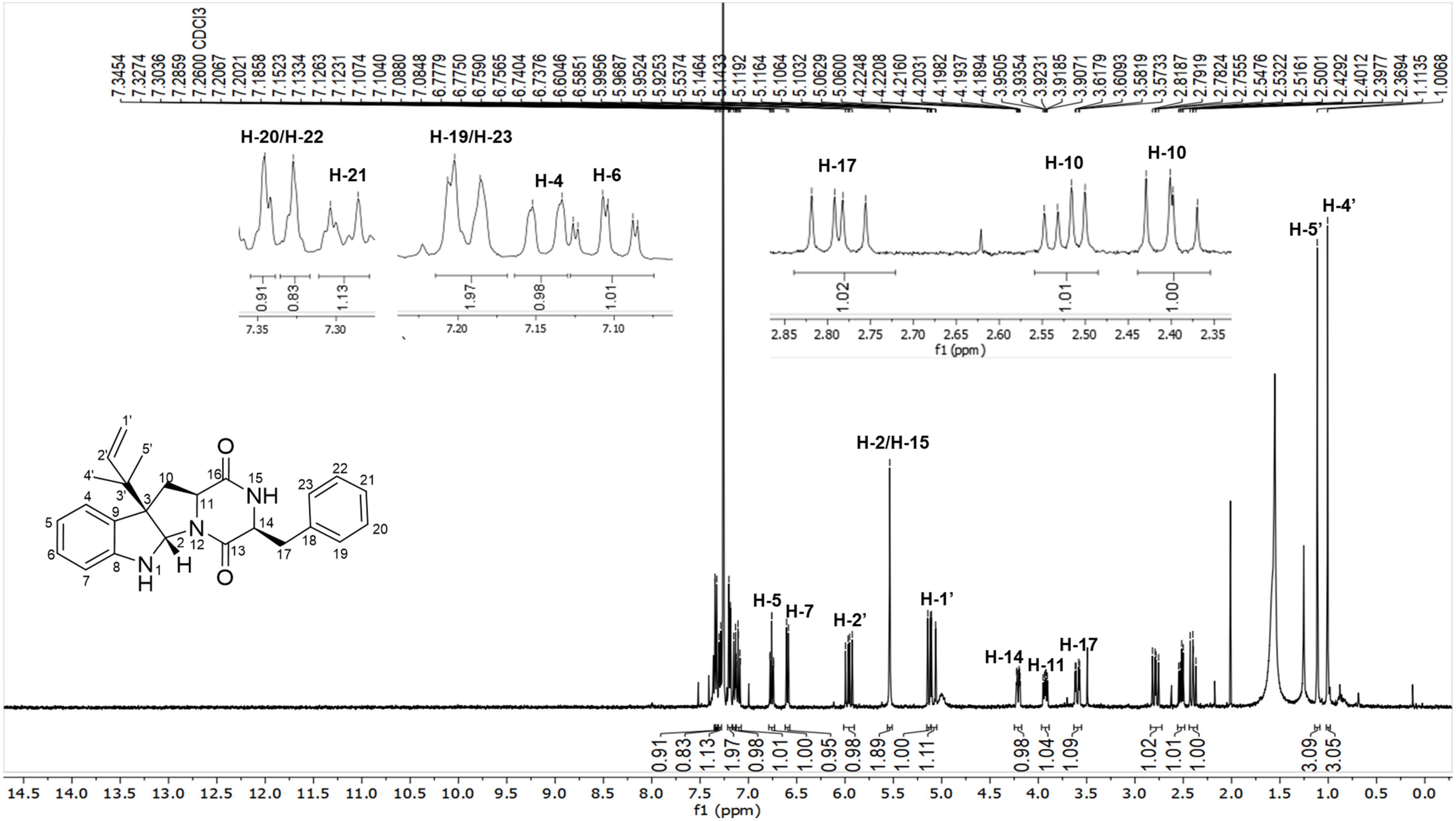

Figure S16. ^{1}H NMR spectrum of **7b** in $CDCl_3$ (400 MHz).

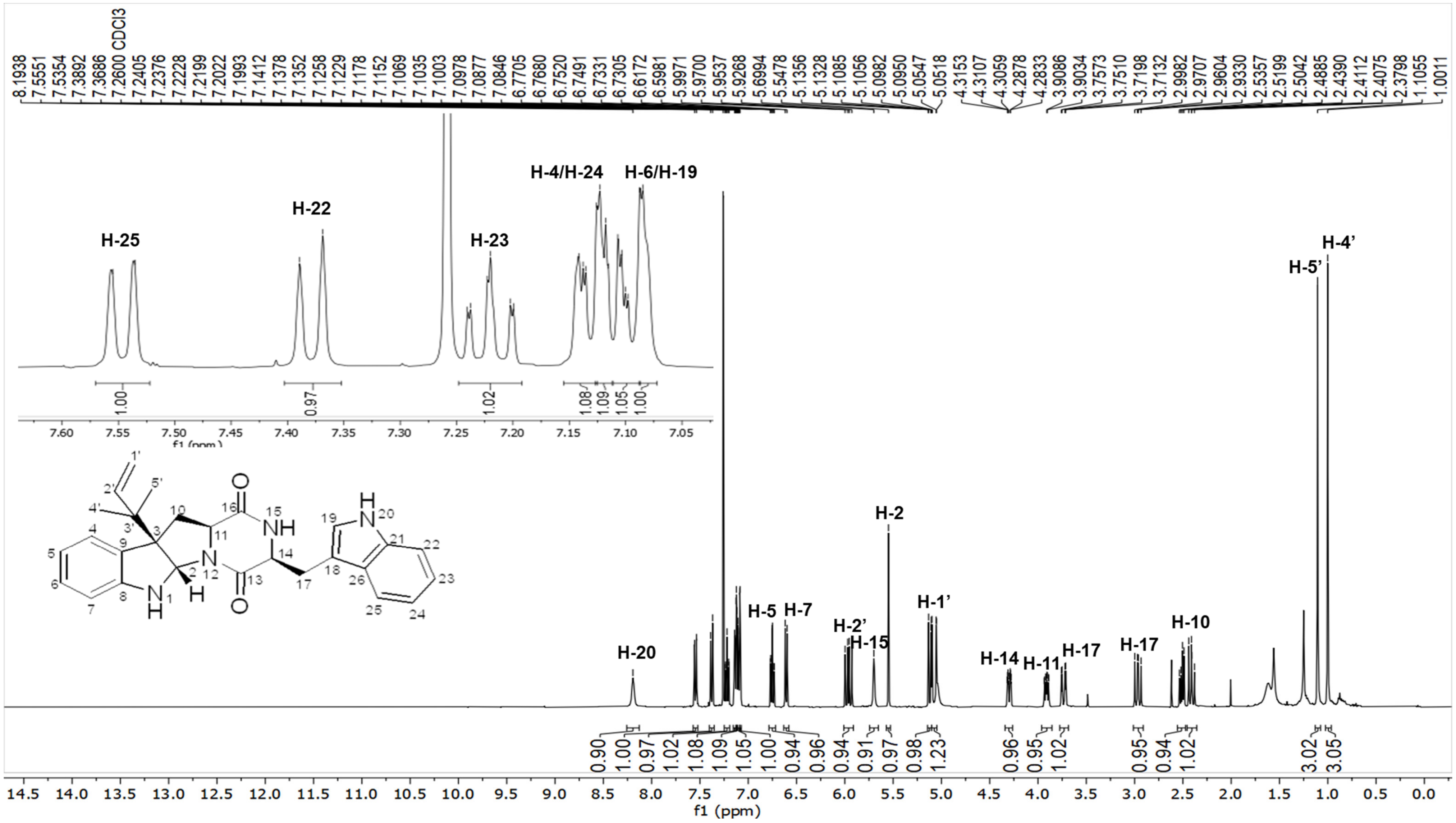

Figure S17. ^{1}H NMR spectrum of **7c** in $CDCl_3$ (400 MHz).

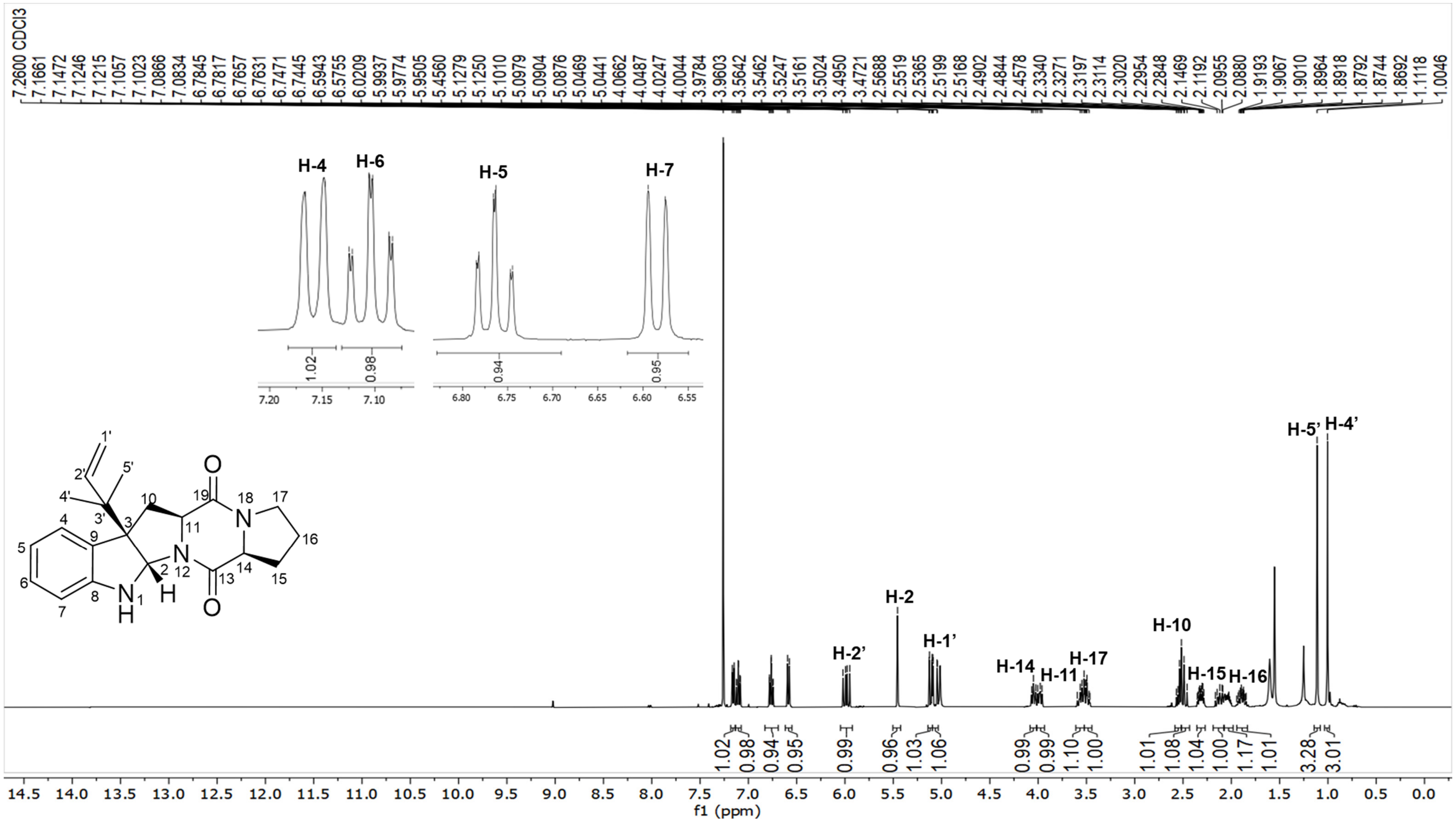

Figure S18. ^{1}H NMR spectrum of **7d** in $CDCl_3$ (400 MHz).

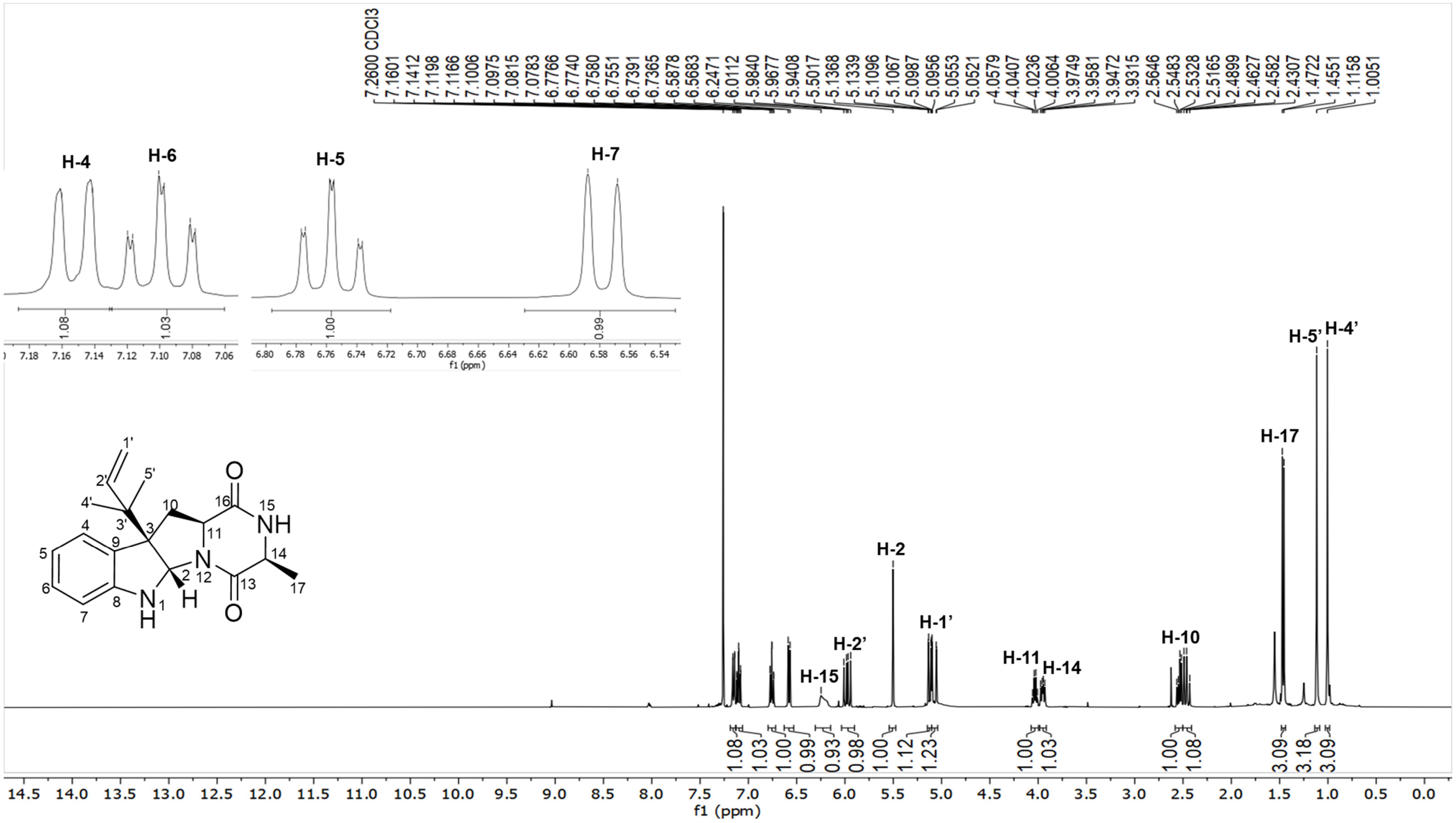

Figure S19. ^{1}H NMR spectrum of **7e** in $CDCl_3$ (400 MHz).

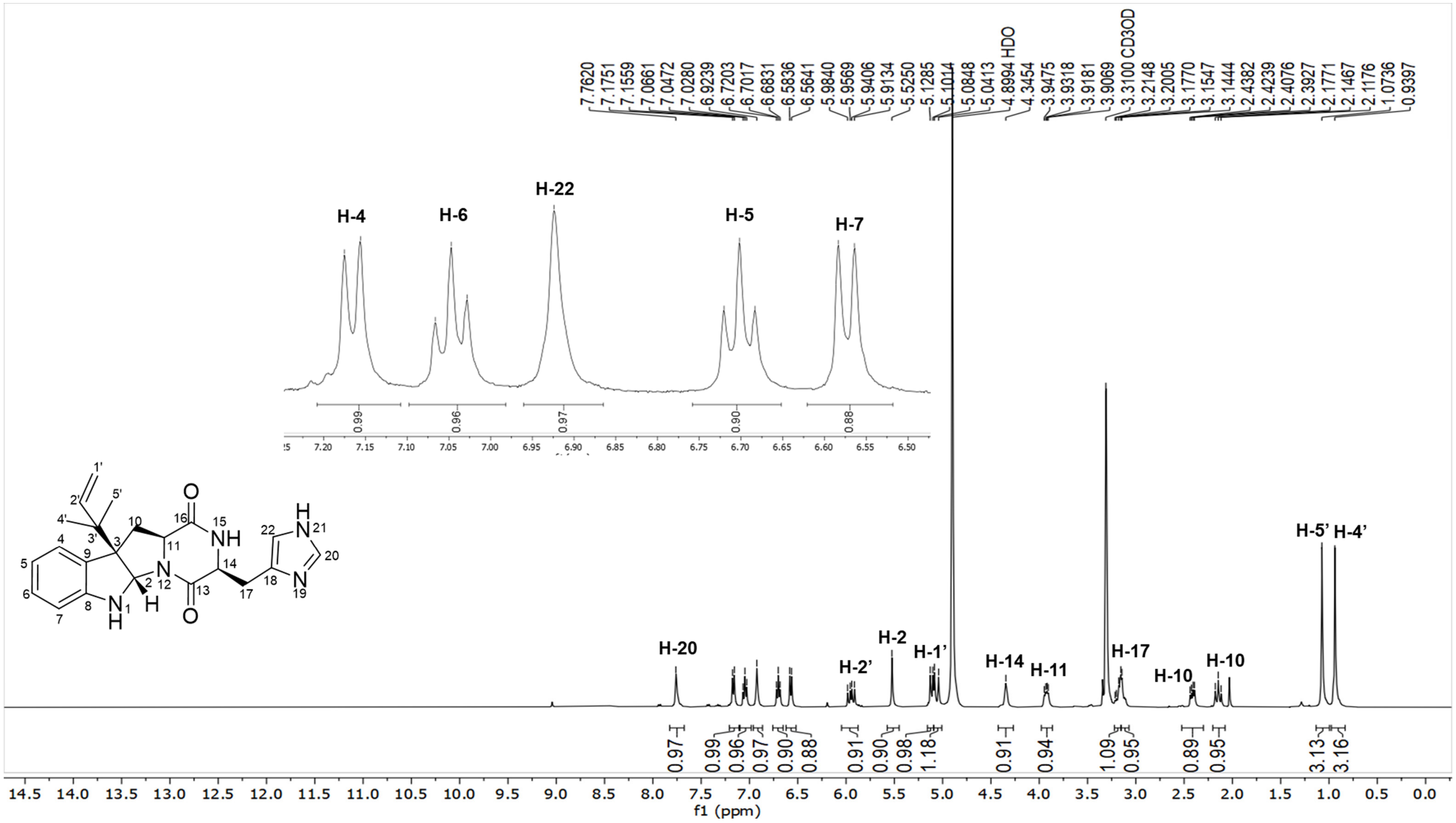

Figure S20. ^{1}H NMR spectrum of **7f** in CD_3OD (400 MHz).

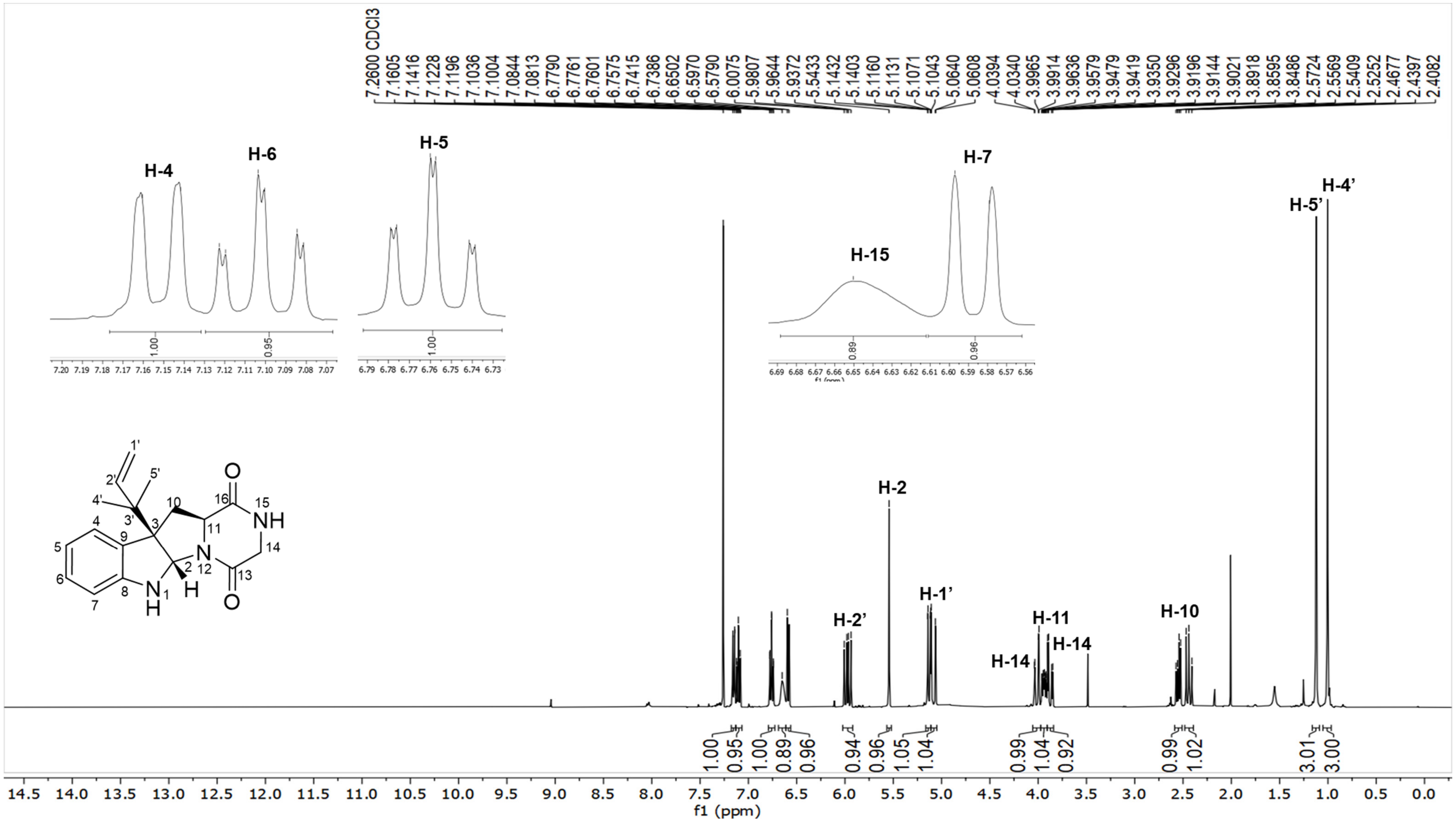

Figure S21. ^{1}H NMR spectrum of **7g** in $CDCl_3$ (400 MHz).

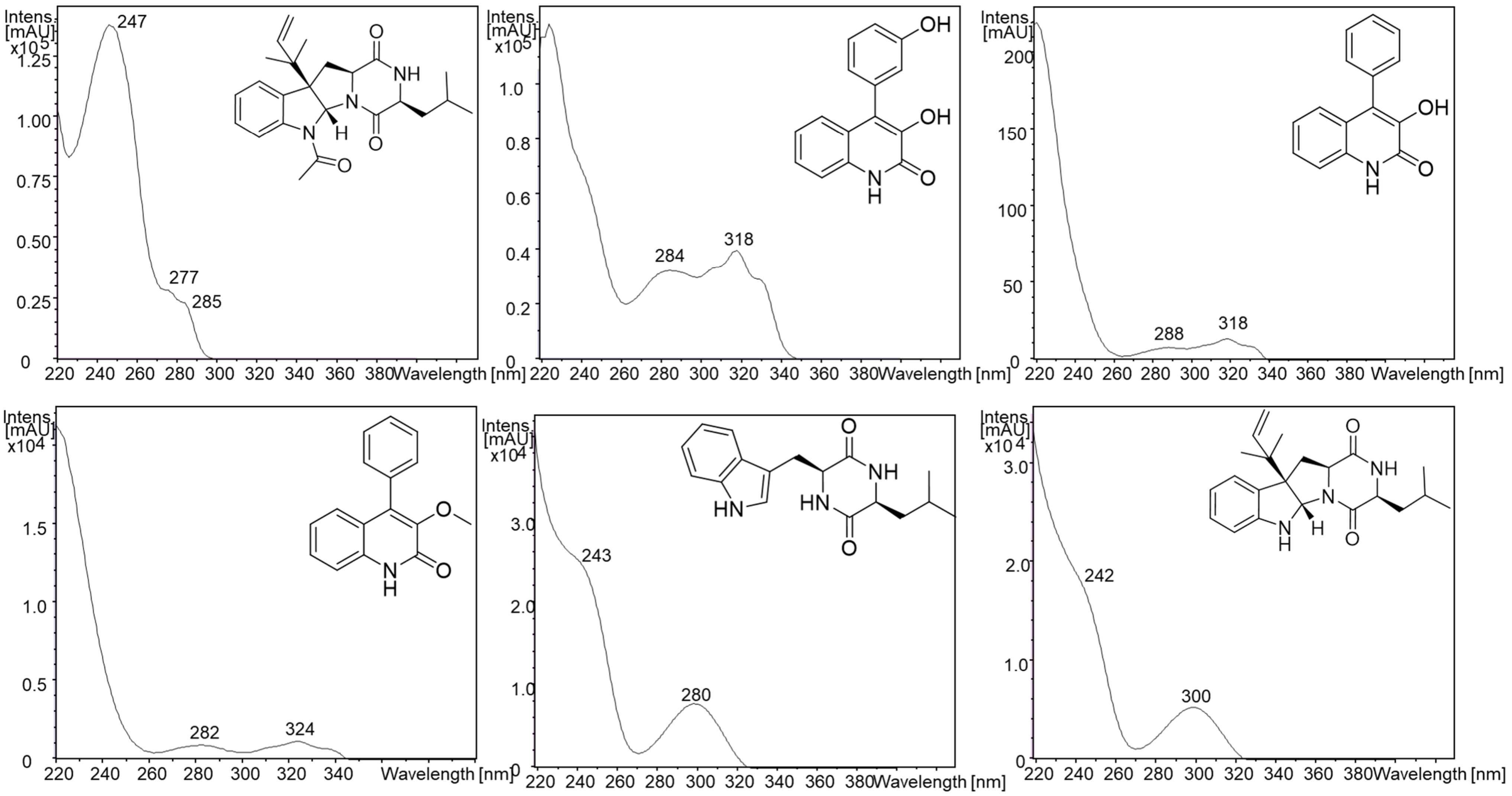

Figure S22. UV spectra of verrucofortine (**1**), viridicatol (**2**), viridicatin (**3**), 3-*O*-methylviridicatin (**4**), *cyclo*-L-Trp-L-Leu (**6**), and *allo*-brevicompanine B (**7**).

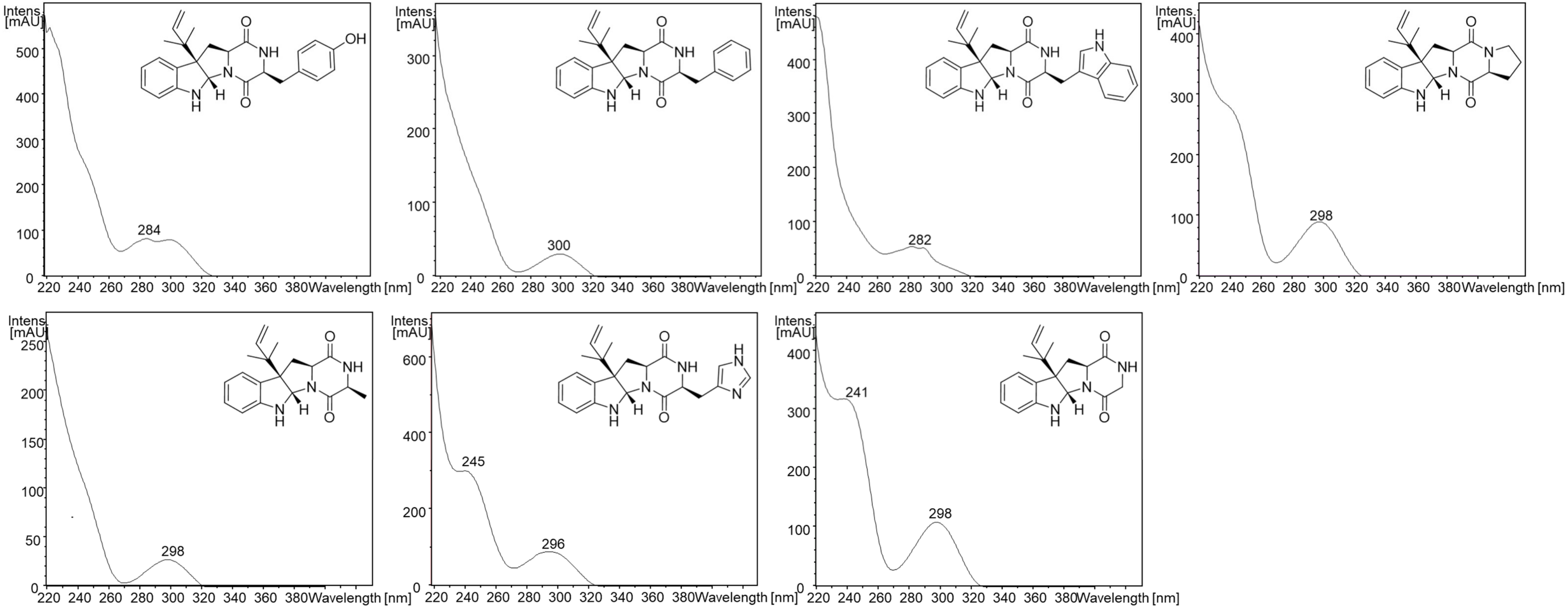

Figure S23. UV spectra of **7a**–**7g**.

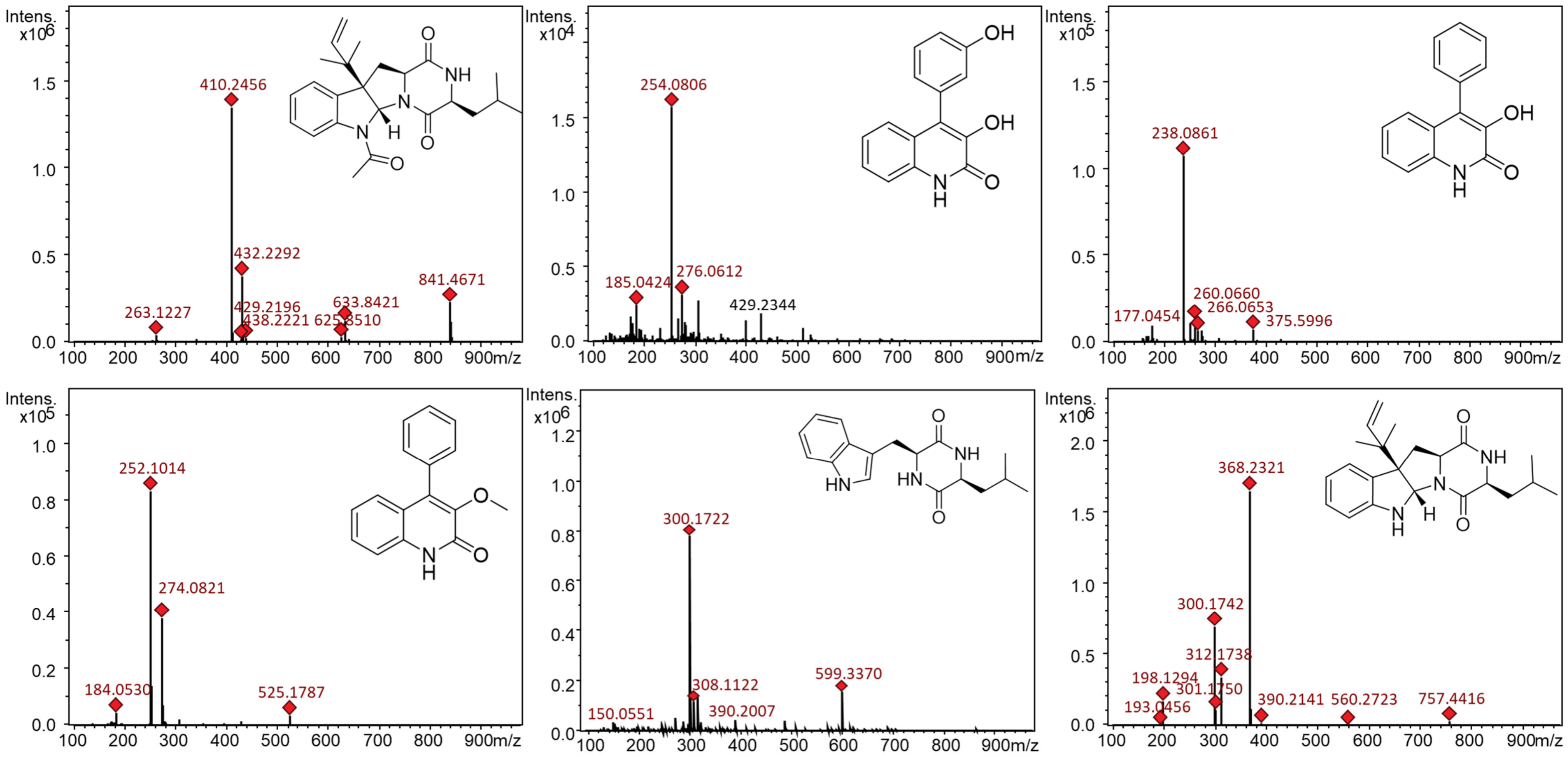

Figure S24. Mass spectra of verrucofortine (**1**), viridicatol (**2**), viridicatin (**3**), 3-*O*-methylviridicatin (**4**), *cyclo*-L-Trp-L-Leu (**6**), and *allo*-brevicompanine B (**7**).

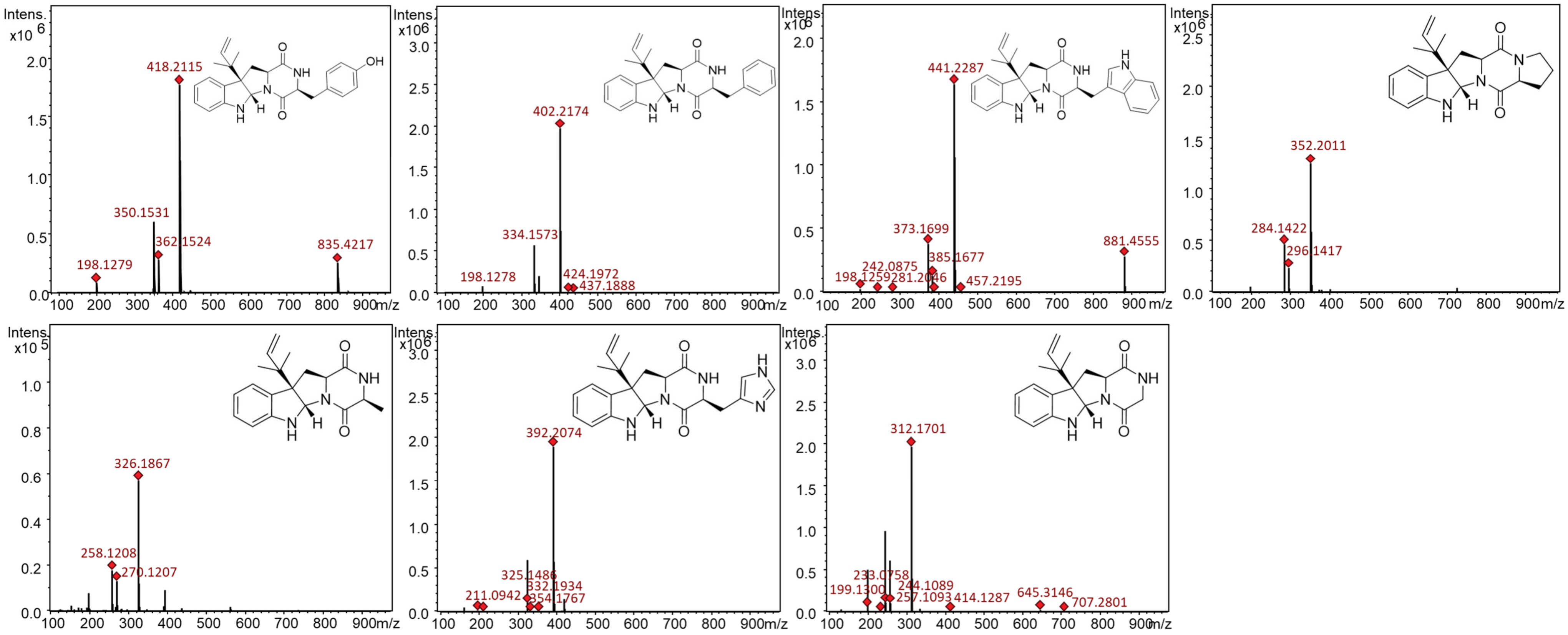

Figure S25. Mass spectra of **7a**–**7g**.

References

(1) Green, M. R.; Sambrook, J. *Molecular cloning: a laboratory manual* 4th; Cold Spring Harbor Laboratory Press, Cold Spring Harbor: New York, 2012.

(2) F. William Studier; Barbara A. Moffatt. Use of bacteriophage T7 RNA polymerase to direct selective high-level expression of cloned genes. *J. Mol. Biol.* **1986**, *189*, 113–130.

(3) Bond, C.; Tang, Y.; Li, L. Saccharomyces cerevisiae as a tool for mining, studying and engineering fungal polyketide synthases. *Fungal Genet. Biol.* **2016**, *89*, 52–61.

(4) Yin, W. B.; Chooi, Y. H.; Smith, A. R.; Cacho, R. A.; Hu, Y.; White, T. C.; Tang, Y. Discovery of cryptic polyketide metabolites from dermatophytes using heterologous expression in *Aspergillus nidulans*. *ACS Synth. Biol.* **2013**, *2*, 629–634.

(5) Fan, J.; Liao, G.; Kindinger, F.; Ludwig-Radtke, L.; Yin, W.-B.; Li, S.-M. Peniphenone and penilactone formation in *Penicillium crustosum via* 1,4-michael additions of *ortho*-quinone methide from hydroxyclavatol to γ-butyrolactones from crustosic acid. *J. Am. Chem. Soc.* **2019**, *141*, 4225–4229.

(6) Wollinsky, B.; Ludwig, L.; Xie, X.; Li, S.-M. Breaking the regioselectivity of indole prenyltransferases: identification of regular C3-prenylated hexahydropyrrolo2,3-bindoles as side products of the regular C2-prenyltransferase FtmPT1. *Org. Biomol. Chem.* **2012**, *10*, 9262–9270.

(7) Yin, S.; Yu, X.; Wang, Q.; Liu, X.-Q.; Li, S.-M. Identification of a brevianamide F reverse prenyltransferase BrePT from *Aspergillus versicolor* with a broad substrate specificity towards tryptophan-containing cyclic dipeptides. *Appl. Microbiol. Biotechnol.* **2013**, *97*, 1649–1660.

(8) Mundt, K.; Li, S.-M. CdpC2PT, a reverse prenyltransferase from *Neosartorya fischeri* with a distinct substrate preference from known C2-prenyltransferases. *Microbiology* **2013**, *159*, 2169–2179.

(9) Wunsch, C.; Zou, H.-X.; Linne, U.; Li, S.-M. C7-prenylation of tryptophanyl and *O*-prenylation of tyrosyl residues in dipeptides by an *Aspergillus terreus* prenyltransferase. *Appl. Microbiol. Biotechnol.* **2015**, *99*, 1719–1730.

(10) Kunizo Arai; Kazutake Kimura; Taisei Mushiroda; Yuzuru Yamamoto. Structures of fructigenines A and B, new alkaloids Isolated from *Penicillium fructigenum* TAKEUCHI. *Chem. Pharm. Bull.* **1989**, *37*, 2937–2939.

(11) Richard P. Hodge; Constance M. Harris; Thomas M. Harris. Verrucofortine, a major metabolite of *Penicillium verrucosum* var. *cyclopium*, the fungus that produces the mycotoxin verrucosidin. *J. Nat. Prod.* **1988**, *51*, 66–73.

(12) Guo, J.; Yang, J.; Wang, P.; Guo, B.; Li, H.; Di Zhang; An, F.; Gao, S. Anti-vibriosis bioactive molecules from Arctic *Penicillium sp*. Z2230. *Bioresour. Bioprocess.* **2023**, *10*, 11.

(13) Shaaban, M.; Sohsah, G. E.; El-Metwally, M. M.; Elfedawy, M. G.; Abdel-Mogib, M. Bioactive compounds produced by strain of *Penicillium sp*. *Int. J. Sci. Eng.* **2016**, *5*, 342–347.

(14) Liu, J.; Yu, H.; Li, S.-M. Expanding tryptophan-containing cyclodipeptide synthase spectrum by identification of nine members from *Streptomyces* strains. *Appl. Microbiol. Biotechnol.* **2018**, *102*, 4435–4444.

(15) Matsumura, Koji, and Takeshi Kitahara. Synthesis of brevicompanines, plant growth regulators. *Heterocycles* **2000**, *55*, 727–733.

(16) Yin, W.-B.; Yu, X.; Xie, X.-L.; Li, S.-M. Preparation of pyrrolo2,3-bindoles carrying a beta-configured reverse C3-dimethylallyl moiety by using a recombinant prenyltransferase CdpC3PT. *Org. Biomol. Chem.* **2010**, *8*, 2430–2438.

(17) Yu, X.; Zocher, G.; Xie, X.; Liebhold, M.; Schütz, S.; Stehle, T.; Li, S.-M. Catalytic mechanism of stereospecific formation of *cis*-configured prenylated pyrroloindoline diketopiperazines by indole prenyltransferases. *Chem. Biol.* **2013**, *20*, 1492–1501.

(18) Yu, X.; Zocher, G.; Xie, X.; Liebhold, M.; Schütz, S.; Stehle, T.; Li, S.-M. Catalytic mechanism of stereospecific formation of cis-configured prenylated pyrroloindoline diketopiperazines by indole prenyltransferases. *Chem. Biol.* **2013**, *20*, 1492–1501.

(19) Ali, H.; Ries, M. I.; Nijland, J. G.; Lankhorst, P. P.; Hankemeier, T.; Bovenberg, R. A. L.; Vreeken, R. J.; Driessen, A. J. M. A branched biosynthetic pathway is involved in production of roquefortine and related compounds in *Penicillium chrysogenum*. *PloS one* **2013**, *8*, e65328.

(20) Ohmomo, S.; Oguma, K.; Ohashi, T.; Abe, M. Isolation of a new Indole alkaloid, roquefortine D, from the cultures of *Penicillium roqueforti*. *Agric. Biol. Chem.* **1978**, *42*, 2387–2389.
